# Improvements in bladder, bowel and sexual outcomes following task-specific locomotor training in human spinal cord injury

**DOI:** 10.1371/journal.pone.0190998

**Published:** 2018-01-31

**Authors:** Charles H. Hubscher, April N. Herrity, Carolyn S. Williams, Lynnette R. Montgomery, Andrea M. Willhite, Claudia A. Angeli, Susan J. Harkema

**Affiliations:** 1 Department of Anatomical Sciences and Neurobiology, University of Louisville, Louisville, Kentucky, United States of America; 2 Kentucky Spinal Cord Injury Research Center, University of Louisville, Louisville, Kentucky, United States of America; 3 Department of Neurological Surgery, University of Louisville, Louisville, Kentucky, United States of America; 4 Department of Urology, University of Louisville, Louisville, Kentucky, United States of America; 5 Frazier Rehabilitation Institute, Louisville, Kentucky, United States of America; Public Library of Science, UNITED KINGDOM

## Abstract

**Objective:**

Locomotor training (LT) as a therapeutic intervention following spinal cord injury (SCI) is an effective rehabilitation strategy for improving motor outcomes, but its impact on non-locomotor functions is unknown. Given recent results of our labs’ pre-clinical animal SCI LT studies and existing overlap of lumbosacral spinal circuitries controlling pelvic-visceral and locomotor functions, we addressed whether LT can improve bladder, bowel and sexual function in humans at chronic SCI time-points (> two years post-injury).

**Study design:**

Prospective cohort study; pilot trial with small sample size.

**Methods:**

Eight SCI research participants who were undergoing 80 daily one-hour sessions of LT on a treadmill using body-weight support, or one-hour of LT and stand training on alternate days, as part of another research study conducted at the Kentucky Spinal Cord Injury Research Center, University of Louisville, were enrolled in this pilot trial. Urodynamic assessments were performed and International Data Set questionnaire forms completed for bladder, bowel and sexual functions at pre-and post-training time points. Four usual care (non-trained; regular at-home routine) research participants were also enrolled in this study and had the same assessments collected twice, at least 3 months apart.

**Results:**

Filling cystometry documented significant increases in bladder capacity, voiding efficiency and detrusor contraction time as well as significant decreases in voiding pressure post-training relative to baseline. Questionnaires revealed a decrease in the frequency of nocturia and urinary incontinence for several research participants as well as a significant decrease in time required for defecation and a significant increase in sexual desire post-training. No significant differences were found for usual care research participants.

**Conclusions:**

These results suggest that an appropriate level of sensory information provided to the spinal cord, generated through task-specific stepping and/or loading, can positively benefit the neural circuitries controlling urogenital and bowel functions.

**Trial registration:**

ClinicalTrials.gov NCT03036527

## Introduction

Loss of urogenital and bowel functions are ranked amongst the most critical consequences of spinal cord injury (SCI), particularly with respect to quality of life [1–4]. Pelvic/visceral organ dysfunction post-SCI has a variety of manifestations including a failure of the bladder to store urine (reduced capacity) and efficiently empty, increased colonic and anal tone resulting in constipation and fecal retention, and impairments in genital responses and sexual arousal including failure to maintain an erection and loss of ejaculation in men [5–8].

While existing therapies aim to manage the prevalent urogenital and bowel issues, interventions addressing recovery of function without adverse side effects are still needed. For example, therapies to improve efficiency of bladder voiding and continence after SCI include clean intermittent catheterization, pharmacologic and surgical interventions [5]. However, catheters are often accompanied by scarring, cystitis and frequent urinary tract infections due to constant introduction of bacteria into the urethra [9]. In addition, frequently used anti-cholinergics have side effects, such as dry mouth and constipation which further complicate the issue of fluid restriction to control timely urine removal [10, 11]. When pharmacological approaches have been proven ineffective, the use of Botulinum toxin A (Botox) injections have demonstrated reductions in incontinent episodes and improved overall bladder compliance in some cases [12, 13]. Even though Botox may have a positive effect on the storage phase, it is always for a limited time (about 6 months), it does not address the voiding dysfunction post-SCI, and it is also associated with increased urinary retention and thus individuals must be able to perform clean intermittent catheterization. Also, more invasive techniques such as surgical implantation of sacral anterior root stimulators requires a posterior rhizotomy resulting in further damage to lower urinary tract afferent pathways leaving the bladder areflexic and also affecting sexual and defecatory reflexes [14, 15]. Therefore, the need for less invasive more tolerable treatment options is critical. Locomotor training (LT), which has been shown to be an effective rehabilitation strategy for improving post-SCI motor outcomes, is one such tool.

Recent discoveries in humans related to activity-dependent plasticity have led to a widely implemented activity-based (generates neuromuscular activation below the level of injury) rehabilitation intervention, LT, for those with incomplete SCI [16–20]. The therapeutic intervention is usually implemented in those with incomplete injuries, even though the mechanistic studies have been done in clinically complete SCI, because while LT optimizes the spinal circuitry, remaining residual supra-spinal inputs may be required to sufficiently excite these networks for successful walking [21]. Generation of locomotion by the interaction of afferent input with central pattern generating networks has been shown in spinally transected animals [22–32] and several of these properties exist in the functionally isolated human spinal cord [33–40]. Motor patterns observed during stepping in individuals with clinically complete SCI [24, 37, 41, 42] are driven by sensory information available to the spinal cord interneuronal networks [33, 39, 43, 44]. Multiple sensory inputs from the periphery during locomotion, particularly limb loading [45] and stepping rate [46] provide information to these networks to improve stepping [41, 43, 47–49]. Thus, activating these lumbosacral spinal circuits chronically might also lead to adaptive changes to other systems such as those controlling urogenital and bowel functions since much of the motor and autonomic output of the spinal cord is driven in large part by afferent input and local or propriospinal circuitry emphasized after SCI conditions [39, 50–53].

Interestingly, our experience with SCI study participants undergoing LT revealed the need for catheterization before each session as a full bladder inhibited stepping. These anecdotal observations suggest an interaction of the locomotor and bladder circuitry that is emphasized after SCI. The interaction of lower limb musculature with the bladder and its sphincter has been observed sporadically over the years, as far back as 1933, in both humans [54, 55] and animals [56, 57]. Flexor and extensor reflexes can be modulated by the state of bladder filling and voiding in both normal individuals and those with CNS damage [55]. In persons with spasticity, the general pattern is that detrusor contractions precede limb flexor spasms [58]. Data from our initial animal studies [59] shows that following 80 one hour per day step training sessions the ability of SCI rats to empty the bladder increased. The mean voiding efficiency (percent volume voided/volume infused) of the LT group was significantly greater than the non-trained group, which was accompanied by a significant increase in the maximum amplitude of bladder contraction and a significantly increased intercontraction interval. It is common for bladder hypertrophy to result from detrusor sphincter dyssynergia in a manner similar to bladder outlet obstruction post-SCI [60, 61]. The sphincter of the LT rats was likely in partial coordination with the bladder to allow for the flow of urine and thus an increase in voiding efficiency.

Given that repetitive activation of this vesico-somatic relationship may be influenced by LT to enhance bladder integrity and function, lower urinary tract function was assessed in a small group of SCI study participants both prior to and after activity-based LT. Due to the close proximity of urogenital and bowel circuitries within the lumbosacral cord, assessments were conducted for bowel and sexual function as well.

## Materials and methods

### Study research participants

A small sample size of eight individuals with chronic SCI, who were enrolled in a LT training study between the years 2012 and 2016 and who met the following inclusion criteria, were directly recruited as a group to participate in this pilot trial: 1) stable medical condition without cardiopulmonary disease or dysautonomia that would contraindicate LT; 2) no painful musculoskeletal dysfunction, unhealed fracture, contracture, pressure sore or urinary tract infection that might interfere with training; 3) no untreated psychiatric disorders or ongoing drug abuse; 4) clear indications that the period of spinal shock is concluded determined by presence of muscle tone, deep tendon reflexes or muscle spasms and discharged from standard inpatient rehabilitation; 5) non-progressive supra-sacral SCI; 6) bladder and sexual dysfunction as a result of SCI; and 7) must be at least 18 years of age. A small group of four individuals who were being evaluated for participation in another study in a parallel time frame and met the inclusion criteria were also recruited. The group of four participants provided values for two assessments with a similar time interval that reflected “usual care”–individuals who conduct their typical daily lives without any study-related change in routine (in the present case, no daily training). This usual care participant group addresses whether there would be any inherent variability between two urodynamic measures and questionnaires with just the long 3-month time-gap, and was not matched to the LT group for gender, lesion level or injury severity as the participants receiving daily training served as their own control (pre- versus post-LT). Based on the recruitment from ongoing parallel studies and inclusion-exclusion criteria, the sample size for this pilot trial was limited to 12 research participants in total (8 LT and 4 usual care).

**Fig 1:**
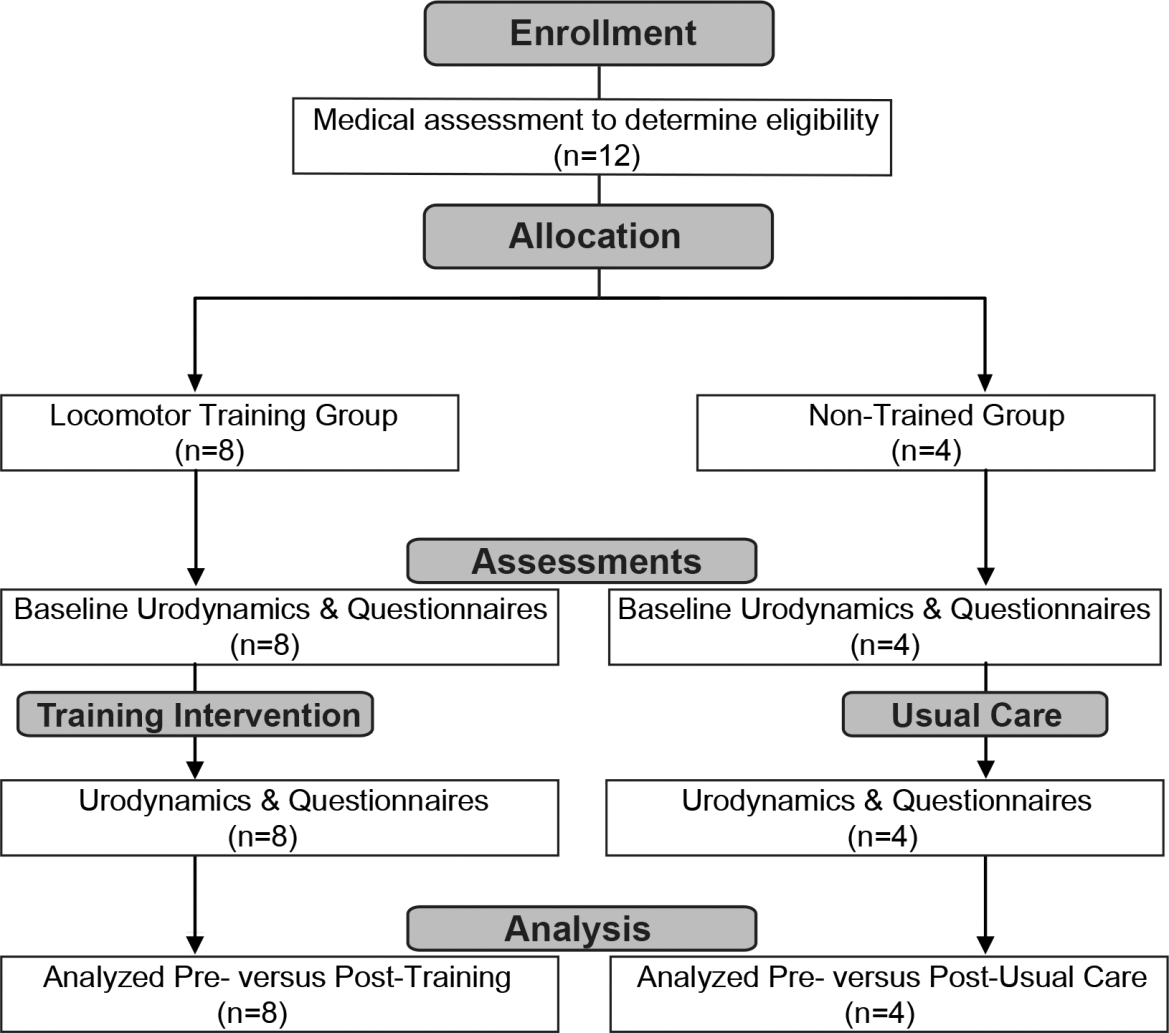
Methods flowchart. Urodynamic and questionnaires (bladder, bowel and sexual function assessments) were conducted after clinical evaluation for eligibility. After approximately 80 locomotor training sessions or usual care (at home in usual routine between assessments for an equivalent time period for 80 training sessions), assessments were repeated. Each participant served as their own control. Individual and group data were analyzed at the conclusion of the study.

All research participants were asked to refrain from taking any bladder medication at least 24 hours prior to urodynamic testing. None of the research participants had received Botox injections prior to or at any point during the study. None of the research participants were on anti-spasticity medication. All procedures were conducted in compliance with NIH guidelines and the protocols reviewed and approved by the Institutional Review Board Committee at the University Of Louisville School Of Medicine. The research participants signed an informed consent for locomotor and stand training, urodynamics, and questionnaire studies. The individuals in this manuscript have given written informed consent (as outlined in PLOS consent form) to publish their case details. A flowchart illustrating the two arms of the study is provided in Fig 1. Recruitment for multiple study groups including those for the current study is ongoing. The authors confirm that all ongoing and related trials for this intervention are registered. Trial registration was done retrospectively as the first research participants were part of a pilot study which did not fit the WHO definition of a clinical trial and subsequent registration was not required in September 2014 by the granting agency.

### Clinical evaluations

Prior to the study, all research participants received a clinical evaluation in order to assess motor and sensory status (Table 1). Two clinicians independently performed the International Standards for Neurological Classification of Spinal Cord Injury [62, 63] in order to classify the injury using the ASIA (American Spinal Injury Association) Impairment Scale (AIS). A physical examination also was performed by a clinician for medical clearance, ensuring participation safety.

**Table 1:** Clinical characteristics of research participants.

**Participant ID**	**Age** Yr*	**Gender**	**Post-Injury** Yr*	**Neuro Level**	**AIS** **Grade**	AIS Score**						**Anal** **Sensation**
						Sensory (T10-S5)			Motor (L2-S1)			
						(score out of 24)			(score out of 25)			
						L LT	L PP	R LT	R PP	L	R	
**A53**	27	M	1.9	T5	A	0	0	0	0	0	0	No
**A57**	26	F	3.5	T4	A	0	0	0	0	0	0	No
**A59**	26	M	2.3	T4	A	0	0	0	0	0	0	No
**A60**	22	M	2.7	T4	A	0	0	0	0	0	0	No
**B23**	31	M	2.11	C5	B	11	8	12	9	0	0	Yes
**C37**	19	F	6.2	T4	C	1	0	0	0	0	0	Yes
**C42**	39	M	2.3	C5	C	3	0	2	2	2	3	Yes
**C43**	29	F	13	C4	D	12	12	16	11	5	15	Yes
**A41 NT**	23	M	6.8	C5	A	0	0	0	0	0	0	No
**A76 NT**	34	M	3.5	C4	A	0	0	0	0	0	0	No
**B21 NT**	30	M	6.7	C4	B	0	6	4	6	0	0	Yes
**C40 NT**	34	M	9.8	C4	C	3	0	5	0	5	1	Yes

Neuro level: neurological level of the lesion; AIS: American Spinal Injury Association (ASIA) Impairment Scale. Sensory score was designated by light-touch (LT) and pinprick (PP) of the left (L) and right (R) lower limb, below the lesion level. Non-trained (NT).

*Age and years’ post-injury at the time baseline CMG testing was performed.

**Results based on the most recent ASIA assessment prior to baseline testing.

All research participants sustained traumatic SCIs.

### Activity-based training

As part of another research study, trained participants received 80 daily sessions of locomotor training (LT) on a treadmill using body-weight support (BWS) (one hour per session) or LT plus stand (weight bearing without stepping) training (one hour of each per day, separated by at least 3 hours) provided by our research team. Suspension was provided by a harness and lift at the minimum support at which limb buckling and trunk collapse was avoided [64]. Manual facilitation was provided to assure dynamic weight bearing equally among the legs and to enhance neuromuscular activation by providing appropriate sensory cues. A trainer was positioned behind the research participant providing assistance at the pelvis and hips while one trainer assisted at each of the legs. Speeds were maintained within a normal speed range for walking (0.89–1.34 m/s). BWS was continuously reduced over the course of the training sessions as the ability to bear weight on the weight bearing limbs improved and manual facilitation was reduced as the ability to step independently improved. The treadmill speeds were varied 25% of the time to challenge the nervous system to adapt to changes in speed (0.5–0.75 m/s). The research participant was encouraged to swing his/her arms in a rhythmical motion with the lower limbs.

Training sessions for standing were conducted according to our published protocol [65]. A custom designed standing frame comprised of horizontal bars anterior and lateral to the research participant was used for upper extremity support and balance assistance as needed. If the knees or hips flexed beyond the normal standing posture, external assistance was provided at the knees distal to the patella to promote extension, and at the hips below the iliac crest to promote hip extension and anterior tilt. Facilitation was provided either manually by a trainer or by elastic cords, which were attached between the two vertical bars of the standing apparatus. Mirrors were placed in front of the research participant and laterally to him/her, in order to allow a better perception of the body position via visual feedback. Research participants were encouraged to maintain standing for 60 min with the least amount of resistance and seated rest periods occurred if requested by the participants.

### Urodynamic investigation

Urodynamic methodology complied with recommendations of the International Continence Society [66]. Assessments were performed by the same registered nurse at pre- and post-training time-points. Prior to filling cystometry, a 12 Fr straight catheter was used to obtain a urine sample from the research participant and a urinary reagent dipstick test (DiaScreen, Arkray) confirmed the absence of bacterial infection. Using the Aquarius LT Urodynamic Investigation system (Laborie Model, Canada), the urodynamic study (UDS) occurred in a dedicated temperature controlled room (22° C) according to established guidelines and standards [67–70]. All participants ceased anti-cholinergic medication 24 hours prior to UDS. The procedure was discussed with the research participant, including any risks and potential side effects not limited to infection and/or bleeding. Cystometry was performed in the supine position via a single sensor, dual channel catheter (7 Fr, T-DOC® Air-Charged™, Laborie, Williston, VT) with continuous filling of sterile, body-temperature water (37° C) at a fixed rate of 20 mL/min. Abdominal pressure was measured via a rectal balloon catheter (7 Fr, T-DOC® Air-Charged™, Laborie, Williston, VT). Pelvic floor electromyography (EMG) (Neotrode II, Laborie, Williston, VT) was recorded using surface patch EMG electrodes and a grounding pad was placed on a bony prominence, usually the hip or knee. Detrusor pressures were calculated by subtracting the intra-abdominal pressure from the intra-vesical pressure. Each research participant was asked to cough to verify intra-abdominal catheter position, was instructed to communicate when s/he first feels a full bladder (first sensation); when s/he first feels the desire to urinate (first urge to void); and when s/he can no longer wait to void (maximum capacity). The volume of water and bladder pressure was recorded. Uninhibited bladder contractions also were identified. The research participant was asked to empty his/her bladder and voiding bladder pressures recorded.

Blood pressure (BP), heart rate (HR) and oxygen saturation were recorded during UDS every minute using an automated sphygmomanometer (DinamapV100; GE Medical Systems, Fairfield, CT). Baseline BP recordings were obtained in the supine position prior to UDS testing. Any signs and self-reported symptoms of AD were documented and observed throughout testing. Bladder filling was ceased if any of the following conditions were observed: 1) spontaneous urine leakage, 2) infused volume ≥ 600 mL, 3) high intravesical pressure ≥ 40 cmH_2_O or 4) AD as evidenced by a sustained systolic blood pressure recording of ≥ 20 mm Hg from baseline and/or intolerable symptoms. At the end of testing, a straight catheterization was performed to quantify residual volumes. A post-fill BP recording was captured to ensure BP values returned to baseline. If AD persisted, it was managed according to established guidelines [71, 72] including sitting the research participant upright to induce an orthostatic BP response, loosening any restrictive clothing, confirming the bladder had been fully emptied, and checking for any other possible sources of triggering stimuli. In this study, no research participants required the use of an antihypertensive agent to manage their AD following the UDS assessment.

### Questionnaires

The International Spinal Cord Injury Data Sets Questionnaires for Urodynamics and Lower Urinary Tract Function (adapted by C.H. Hubscher to include average number of nightly bladder emptying/day) [73, 74], Bowel Function (adapted by C.H. Hubscher to include an expansion of the average time required for defecation) [75, 76], Female Sexual and Reproductive function (adapted by C.H. Hubscher to include the 19-item Female Sexual Function Index (FSFI) that is divided into six domains–desire, arousal. Lubrication, orgasm, satisfaction and pain [77, 78]) as well as Male Sexual Function (adapted by C.H. Hubscher to include the 15-item International Index of Erectile Function (IIEF) that is divided into five domains–erectile function, orgasmic function, sexual desire, intercourse satisfaction, and overall satisfaction [79, 80]) were administered at pre- and post-training time points to assess the impact of SCI on overall bladder, bowel and sexual function management and health.

### Data acquisition and statistics

Data was acquired during UDS from the first cystometrogram recording in each research participant at pre- and post-training time points. Bladder capacity was calculated as the volume of leaked or voided water plus any residual amount removed from the bladder. Voiding efficiency (VE) was calculated as: VE = volume voided / (volume voided + residual) X 100. Compliance was calculated by dividing the volume change (ΔV) by the change in detrusor pressure (ΔPdet) during that change in bladder volume and was expressed in ml/cm H_2_O [81]. Note that the ICS recommends that two standard points should be used for compliance calculations: the detrusor pressure at the start of bladder filling and the corresponding bladder volume (usually zero), and the detrusor pressure (and corresponding bladder volume) at cystometric capacity or immediately before the start of any detrusor contraction that causes significant leakage, which affects bladder volume and overall compliance values [70]. Low compliance, according the International Spinal Cord Injury Data Sets is considered to be at <10 cm/H_2_O [73]. The intravesical pressure (Pves) at which involuntary expulsion of water/urine from the urethral meatus was observed was considered the leak point pressure (LPP). Maximum detrusor pressure (MDP) was identified as the highest Pves during the voiding phase of the CMG.

Analyses were performed using SPSS v19−20 (IBM, North Castle, NY). Binomial proportion tests were performed to assess the distribution of participants (compared to random occurrence) showing improvements in bladder capacity, leak point pressures, voiding efficiency and the area of detrusor contraction. All urodynamic parameters were normally distributed as assessed by the Shapiro-Wilk test. Therefore, the Student’s paired t-test was used to analyze to collective pre/post differences. All data are expressed as mean ± SD (standard deviation). Significance value was at p ≤ 0.5.

## Results

Clinical characteristics of the eight research participants receiving approximately 80 one hour training sessions is provided in Table 1, including each research participant’s AIS scores as assessed just prior to participation in the study. The research participants had a mean age of 27.4 years at the start of training (range of 19 to 39 years) and 62.5% (5 of 8) were male. Five had a thoracic (T4 or T5) neurological level of injury, with the other three being at cervical levels (C4 or C5). From the date of the initial baseline urodynamic assessment, the mean time post-injury was 4.4 years. Adaptations of the International SCI Basic Data Sets were used pre- and post-training for assessing bladder function, which was a combination of the lower urinary tract function (LUT) data set [74] and the urodynamic data set [73], bowel function [75, 76], and male [79, 80] and female [77, 78] sexual function. The mean time interval between questionnaires/cystometry and completion of the one hour daily training sessions was 97 days. All research participants were also asked to complete weekly voiding diaries (2011 American Urological Association Foundation, Inc.), but there is an insufficient amount of data to report due to low compliance (1 out of 8 participants).

### LUT function data set questionnaire

Questionnaires completed just prior to and at the conclusion of 80 training sessions included items such as urinary tract impairments unrelated to the spinal lesion, awareness of need to empty the bladder (normal, indirect or none, which was adapted to include localization of the indirect sensation), bladder emptying method and frequency, incontinence frequency (daily, weekly, monthly, none) and medication usage. Note that mean times per day to empty was also adapted to separate out the incidence of nocturia, the need to wake one or more times at night to void. The results are summarized in Table 2. Note the changes in frequency of nocturia and incontinence post-training for several research participants. Interestingly, one AIS A participant, who stated at baseline (pre-training; 2 years post-injury) that clean intermittent catheterization was conducted solely by time (reported having no sensations even on occasions with catheterizations as high as 1000 ml), described having chills down both legs after undergoing LT (change noted starting after 30 sessions [approximately 6 weeks] of LT). This sensation was also reported just prior to void during the post-train cystometry session (not so at baseline).

**Table 2:** LUT elements adapted from international SCI data set [74].

Data Set Function		Pre-Training	Post-Training
Urinary tract impairment unrelated to spinal lesion		0%	Unchanged
Awareness of need to empty bladder		88% indirect (Ф: 1, AIS A)	100% indirect
Indirect localization site	Lower Pelvis/Abdomen	AIS A = 33% AIS B-D = 100%	AIS A = 50% AIS B-D = 100%
	Above lesion level only	28.5% (AIS A)	12.5% (AIS A)
Bladder emptying method (n = 8)		75% CIC; 12.5% SP; 12.5% Monti [82]	Unchanged
Bladder emptying	Waking hours: mean #	5.0 ± 1.4	5.0 ± 0.8
	Sleeping hours: nocturia	57% participants	28.5% participants
Involuntary urine leakage (incontinence)		62.5% participants	37.5% (2 of 3 reported reduced leakage)
Anticholinergic medications for bladder		75% participants	Unchanged

AIS, ASIA (American Spinal Injury Association) Impairment Scale; CIC, Clean Intermittent Catheterization; LUT, Lower Urinary Tract; Monti procedure–based upon Mitrofanoff principle; SP, Suprapubic indwelling catheter; Ф, Non-Responder

### Urodynamic assessments

**Fig 2:**
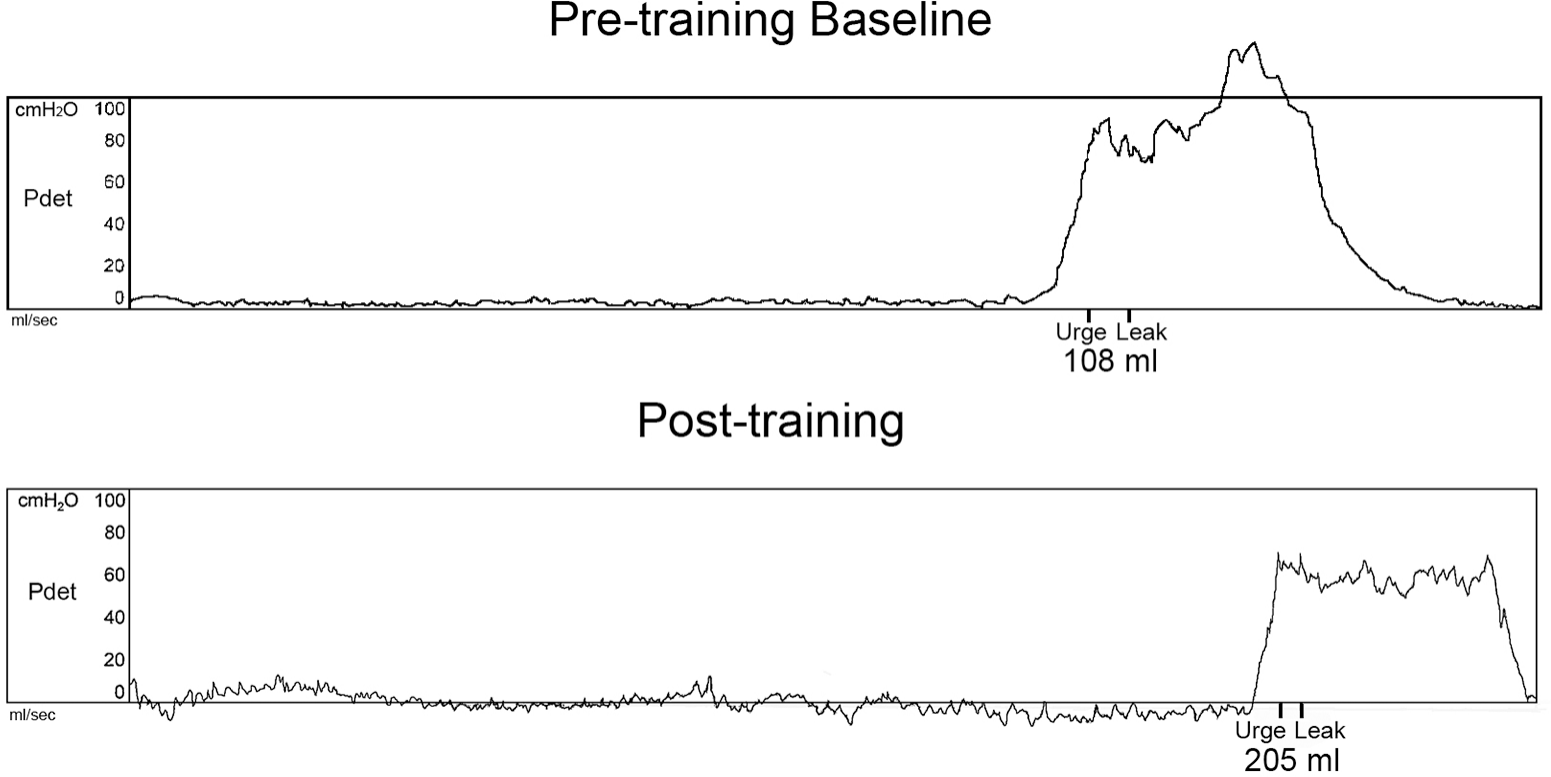
Detrusor pressure recording example. Raw recording of detrusor pressures (Pdet) from research participant A57 for the initial fill cycle pre-training and post-training. Note that the urge sensation precipitated by the rise in the bladder pressure immediately preceded the onset of leak, which occurred at almost twice the fill volume post-training. On the pre-training fill/void cycle, the leak occurred at 05:54 (min:sec), while post-training the leak occurred at 10:33 (20 ml/min–constant fill rate).

**Fig 3:**
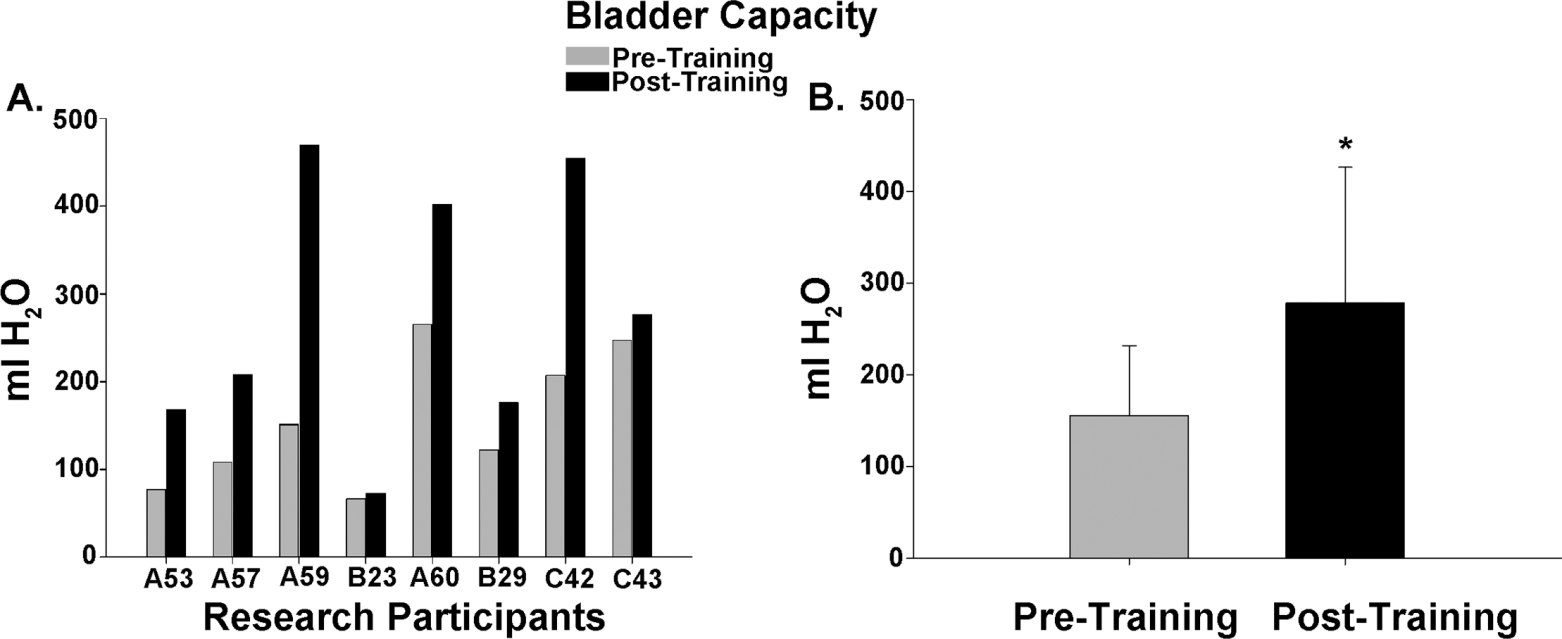
Bladder capacity data summary. Bladder filling ceased and capacity measured (leak + residual volumes) with the occurrence of either spontaneous urine leakage (n = 5; reflex void), autonomic dysreflexia (n = 2; Participants A60 and C42) or a voluntary void following a strong urge (AIS D Participant C43). (A) A comparison of pre- and post-training bladder capacity values in each of the eight participants. A binomial proportion test indicates that a significant majority of the research participants demonstrated an improvement in bladder capacity (vs. random occurrence, p < .05). Note that research participant B23 had a suprapubic catheter and thus, the increase in capacity was incremental. (B) Bladder capacity increased significantly post-training (p = .02, 155.4 ± 76.1 vs 278.5 ± 147.8 ml).

**Fig 4:**
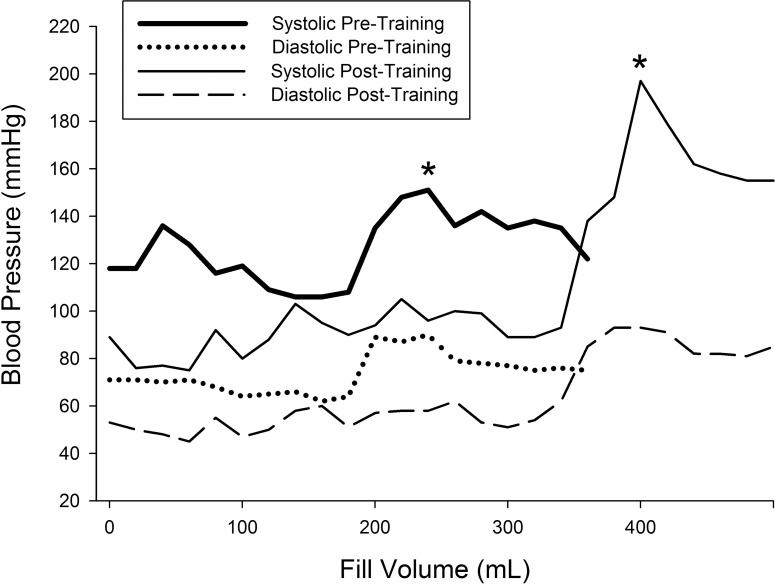
Blood pressure recording example during bladder filling. Blood pressure recordings for research participant C42 during filling pre- and post-training. Filling was stopped at the onset of autonomic dysreflexia (increase of > 20 mmHg), as indicated by the asterisk (*). Note that the shift to a higher volume post-training is indicative of an increase in bladder capacity. Blood pressure returned to baseline by the time the bladder was emptied by catheterization for residual volume measurement.

**Fig 5:**
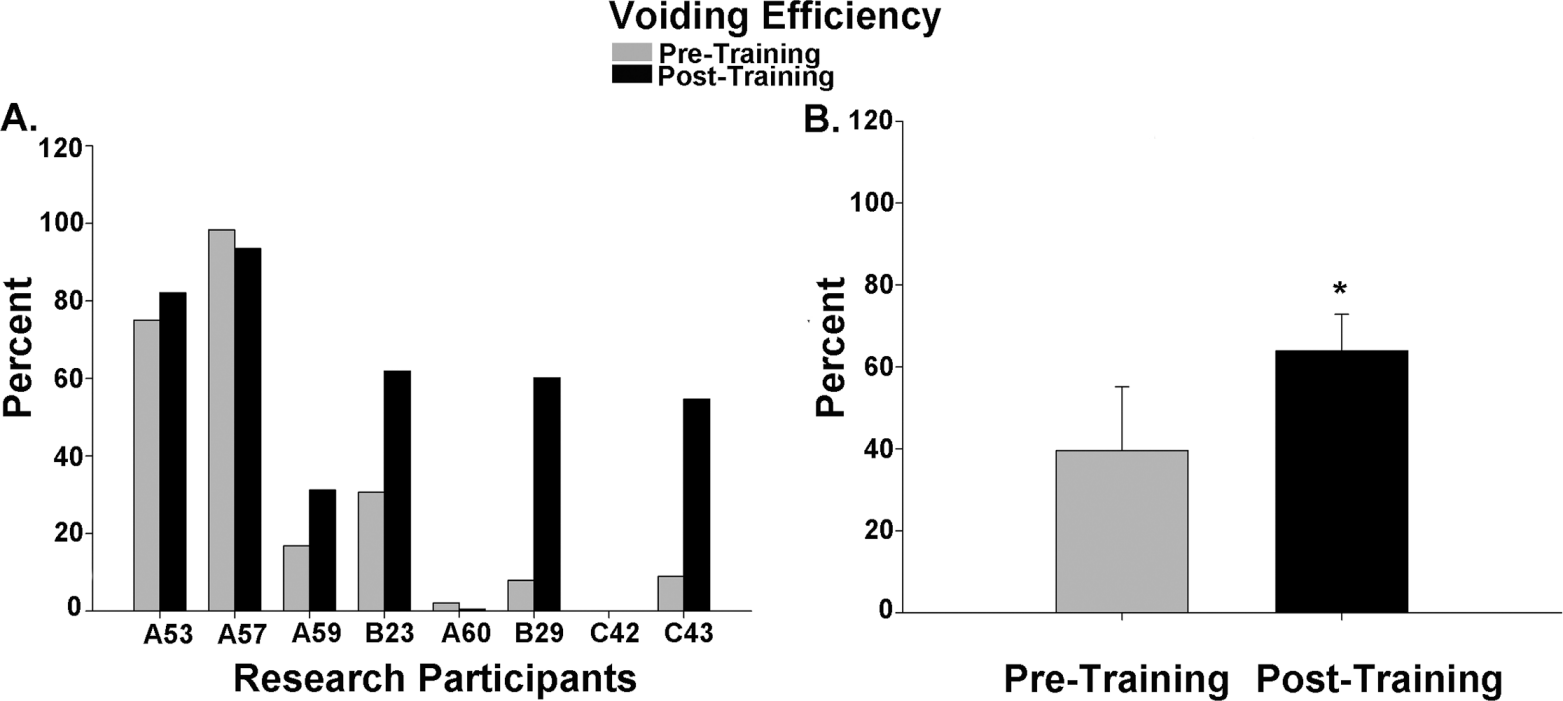
Voiding efficiency data summary. A comparison of pre- and post-training voiding efficiency (VE) values in the eight research participants. While the majority of participants demonstrated an improvement in VE, participant C42 did not have a leak/void and participant A60 just leaked a few drops. When considering the six research participants that had a measurable efficiency, a significant improvement in VE values occurred collectively post-training (p = .046; 39.6 ± 15.5% vs 63.9 ± 8.9%).

**Fig 6:**
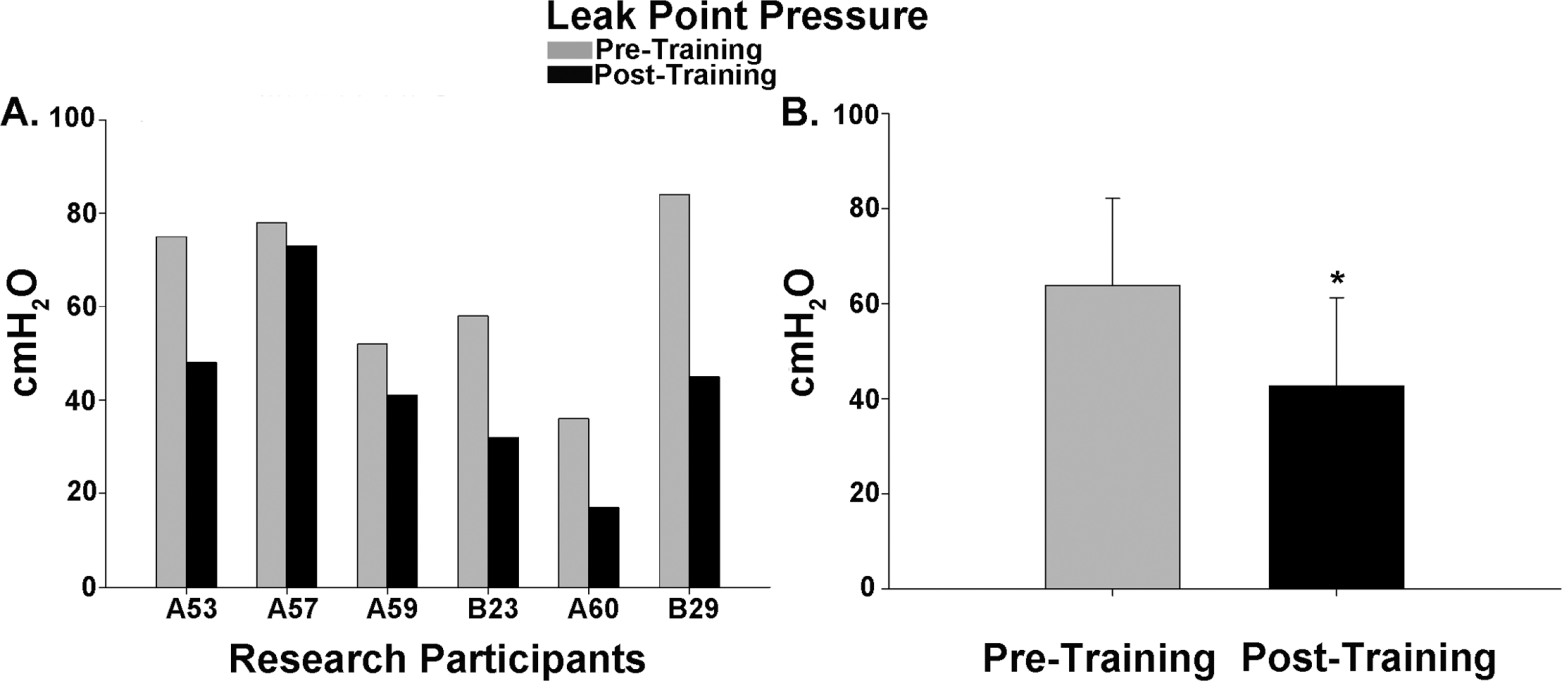
Leak point pressure data summary. Intravesical bladder pressure values at the onset of leak/void in six of eight research participants during cystometry. (A) A comparison of pre- and post-training bladder leak pressure values in each of the six participants. A binomial proportion test indicates that a significant majority of the research participants demonstrated an improvement in overall bladder leak point pressure (vs. random occurrence, p < .05). (B) The intravesical pressure at the time the leak/void was recorded was significantly reduced post-training (p < .01, 63.8 ± 18.3 vs 42.7 ± 18.6 cmH_2_O).

Cystometry was conducted just prior to training and repeated again just after completion of the 80 LT training sessions. An example of the detrusor pressure recordings from one research participant is provided in Fig 2. Note that the pressures were maintained at low levels post-training despite the increase in capacity, which was significantly increased for the group of 8 research participants. A summary of the capacity data is illustrated in Fig 3 histograms (Fig 3A–individual participant data; Fig 3B–mean pre/post LT data). For two of the 8 research participants (A60 and C42), capacity was reached based upon the occurrence of autonomic dysreflexia (a sustained systolic blood pressure recording increasing ≥ 20 mm Hg from baseline with intolerable symptoms [83, 84]). An example from C42 is provided in Fig 4. Note that A60 only leaked a few drops at this fill level, whereas C42 did not leak at all, and thus had a voiding efficiency of zero (amount of leak/void relative to total of leak/void plus residual). Voiding efficiencies for all research participants (pre- and post-training) are provided in Fig 5. For the six research participants that had a void (involuntary leak for five of six), the detrusor leak point pressure was also assessed as pressures above 40 cmH_2_O are considered abnormal and at risk for upper urinary tract deterioration [11]. Overall as a group, there was a 35% decrease in leak point pressure from pre-training to post-training time-points. A summary of the leak point pressure data is illustrated in Fig 6 histograms (Fig 6A–individual participant data; Fig 6B–mean pre/post LT data).

**Fig 7:**
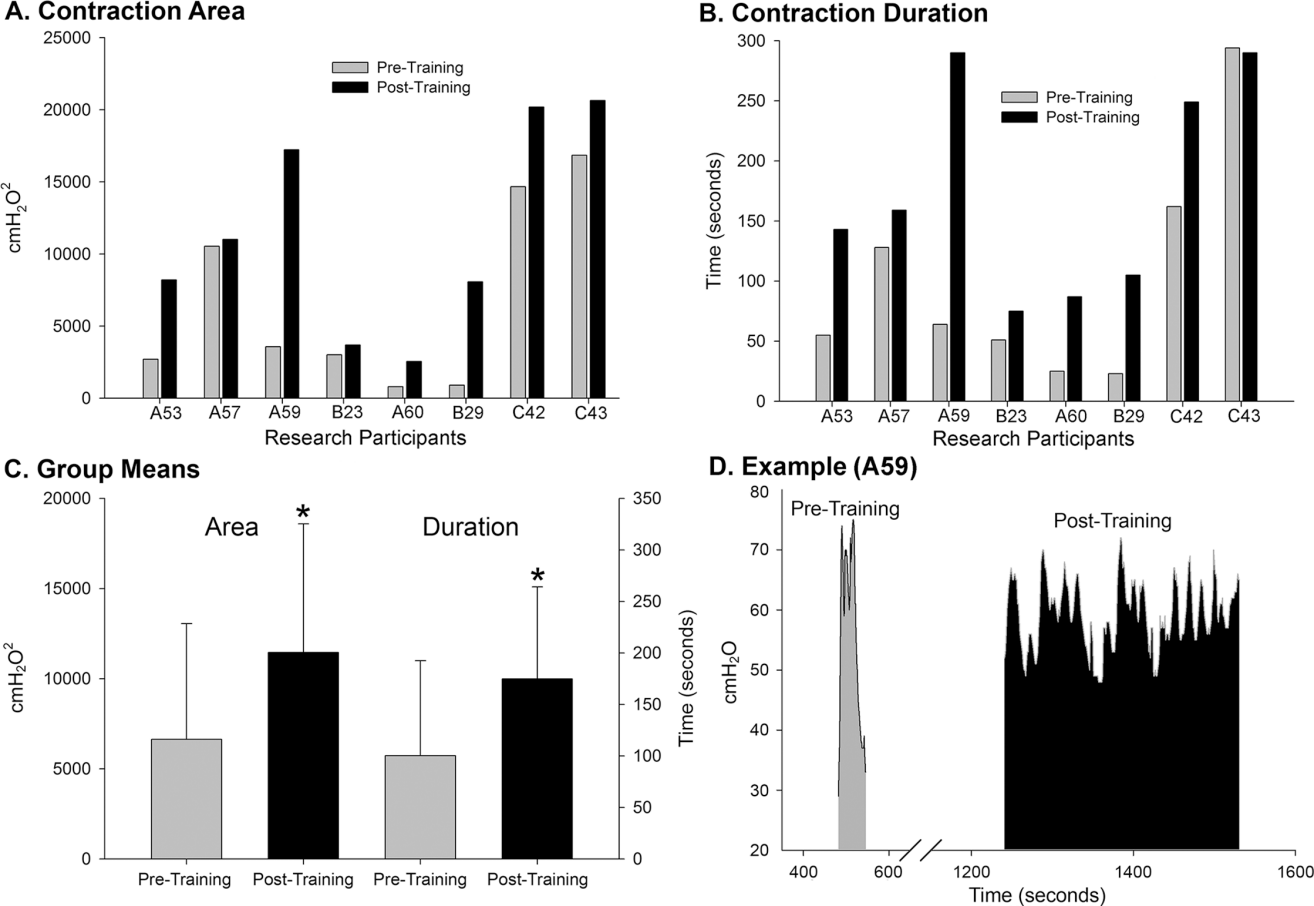
Bladder contraction data summary. A comparison of pre- and post-training bladder contraction values (area under the curve and duration) for the eight research participants receiving LT. All research participants had an improvement in the detrusor contraction area (**A**) and 7/8 had improved duration (**B**). Collectively, the group values post-training (**C**) were significantly greater compared to pre-train values (area—p = .016; duration–p = .019). An example of the entire detrusor muscle contraction cycle is provided for participant A59 (**D**) to illustrate a greater capacity for bladder filling was maintained post-training and generated a larger voiding contraction for a longer duration.

**Fig 8:**
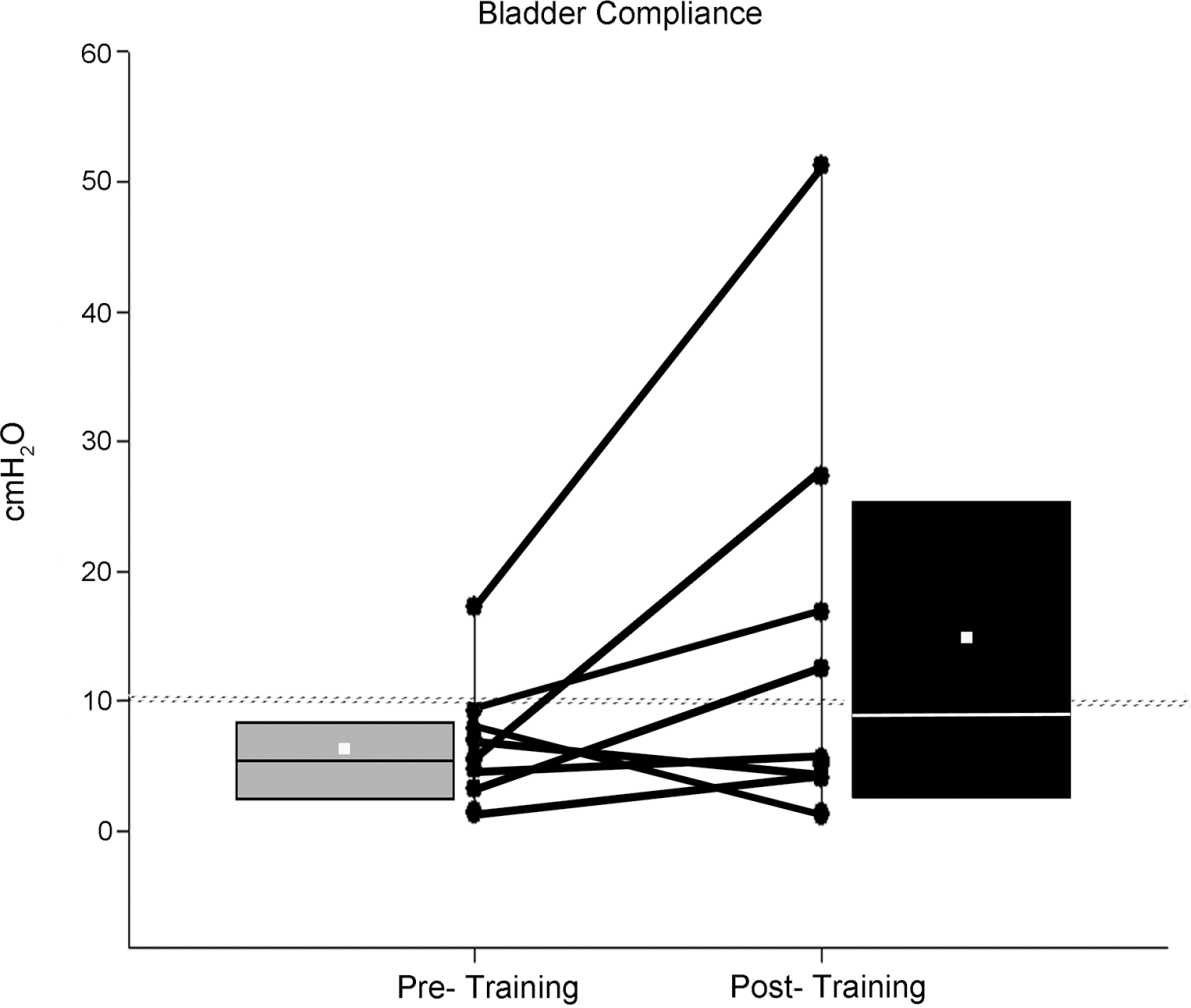
Bladder compliance data summary. Most research participants (85%) had low compliance (<10 cmH_2_O - ΔP/ΔV) at the pre-training baseline cystometric recording. However, post-training, 50% showed an improvement in bladder filling under lower pressures. A ranking and average of the compliance values at the pre-training time point indicate a median (horizontal line) and mean (small white square) value of 6.3 and 7.0 ± 4.8 cm/H_2_O, respectively, while post training, the median and mean values were 9.0 and 15.5 ± 16.8 cm/H_2_O, respectively (p>.05).

Further analysis of the cystometry data included quantification of the size of the bladder contraction (measurement of area under the curve), the duration of the contraction, and assessment of bladder compliance (change in volume divided by change in detrusor pressure). The pre- versus post-training data showing significant changes are provided in Figs 7 and 8. In addition, there were no differences in fill volumes at first sensation pre- versus post-training and no differences in the maximum detrusor pressure (64.9 ± 34.8 cmH_2_O pre-training versus 59.6 ± 30.4 cmH_2_O post-training). Interestingly, there was a gender effect for fill volumes at first sensation (male participants 216.5 ± 78.4 ml versus female participants 57.5 ± 55.9 ml [SD]; p = .003), a pattern which has been previously found following cystometry in non-injured, healthy (no urological abnormalities) individuals [85].

### Bowel function data set questionnaire

**Fig 9:**
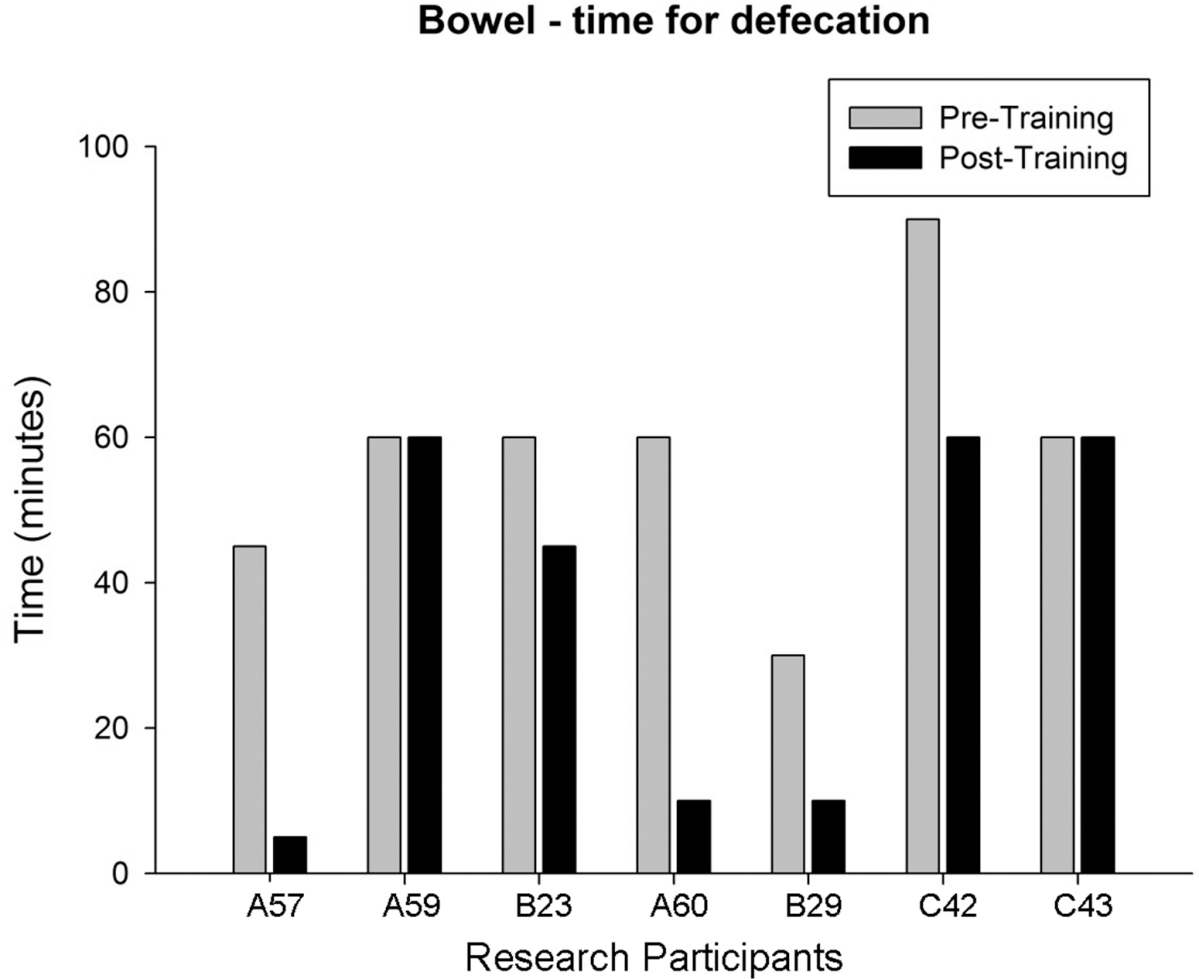
Participant estimated time for defecation. Average time for defecation over “last four weeks” for seven of eight research participants (bowel data set used beginning with participant A57). Pre-/post-training group means were 57.9 ± 18.2 and 35.7 ± 26.2, respectively. A binomial proportion test indicates that a significant majority of the research participants demonstrated an improvement in bowel function (vs. random occurrence, p < .05).

Questionnaires completed just prior to and at the conclusion of 80 training sessions included items such as awareness of need to defecate, defecation method, time required and frequency of defecation, incontinence frequency, and medication usage [75, 76]. The results are summarized in Table 3. Note the change in frequency of fecal incontinence as well as the significant decrease in time required to defecate pre- versus post-training. Individual defecation time data (question asks for an estimated average “within the last four weeks”) is provided in Fig 9.

**Table 3:** Bowel items from international SCI data set [75, 76].

Data Set Function		Pre-Training	Post-Training
GI or anal sphincter dysfunction unrelated to spinal lesion (n = 7)		0%	Unchanged
Awareness of need to defecate		57% indirect (Ф: 3, AIS A)	71% indirect
Indirect localization site	Rectal pressure	75% (AIS C-D)	60%
	Above lesion level only	0% (n = 4)	0% (n = 5)
Bowel emptying method (n = 8)		75% Suppository; 25% Enema	Unchanged
Time for Defecation		57.9 ± 18.2	35.7 ± 26.2*
Frequency of fecal incontinence (n = 7)		43%, monthly	0%
Frequency of defecation (n = 8)		37.5% Daily; 62.5% EOD	25% Daily; 75% EOD
Medication usage for bowel (n = 7)		0%	Unchanged
Oral laxative usage		43% (n = 7)	37.5% (n = 8); (half amount for n = 2/3)

*Significant change from pre-training, p = .022 (±SD); EOD, every other day; Ф, Non-Responders.

### Sexual function data set

**Fig 10:**
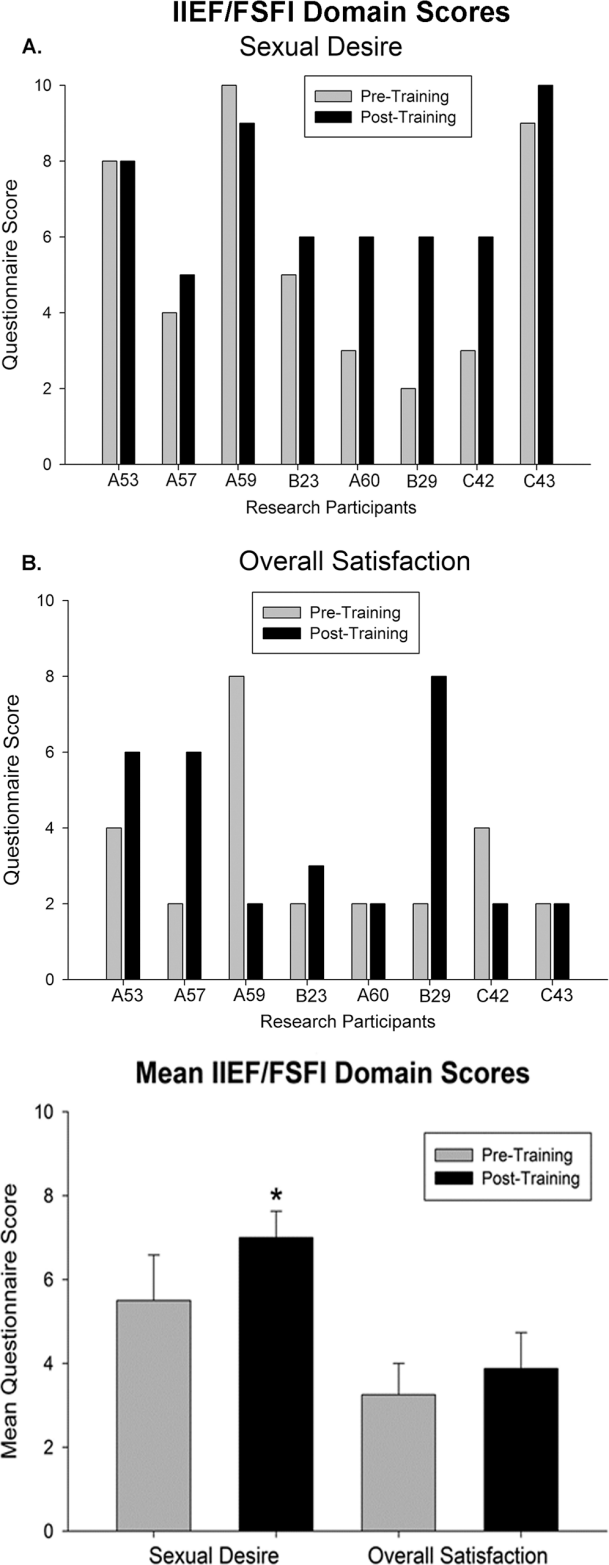
Summary of sexual function data. Sexual function domain scores for desire and satisfaction (total score from two 5 point questions). Group means are provided in the bottom histogram. Whereas scores for desire significantly increased post-training (*, p = .04), overall satisfaction remained unchanged. Note that scores above the two point minimum for satisfaction were given when research participants reported engagement in sexual activity.

Widely-used and validated questionnaires, either the International Index of Erectile Function or the Female Sexual Function Index, were completed by all male and female research participants. Additional data was collected on medication usage for sexual function. No sexual activity was reported on questionnaires for six research participants for at least one of the two time points, leaving primarily the sexual desire and overall satisfaction domains for analysis (common elements in both male and female surveys). The results are presented in Fig 10. For males, Question 15 of the IIEF (5 points) and the 4 point Erectile Hardness Grading Scale (EHGS) were used specifically for erectile function [80]. No significant differences were found post-training relative to baseline (Question 15 re confidence to get and keep an erection—pre-training: 2.0 ± 0.41and post-training: 2.5 ± 0.50; EHGS: pre-training 2.6 ± 0.40 and post-training: 3.2 ± 0.37). Only one of the five male research participants reported using Viagra (50 mg pre- and post-training) and all five reported being unable to ejaculate at both study time-points.

### Usual care participant data

**Fig 11:**
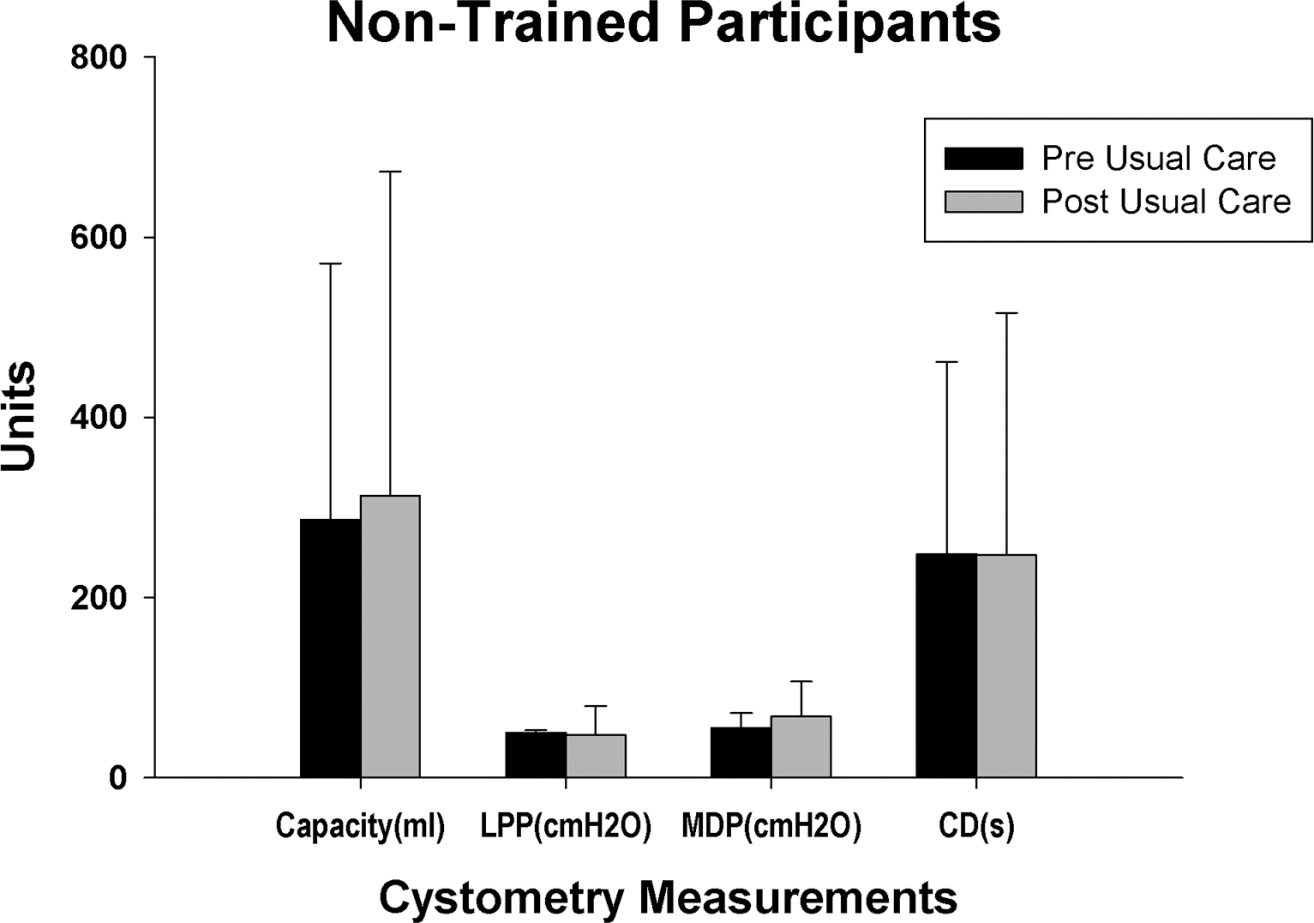
Summary of cystometry data for usual care participants. Summary of cystometry results for the four non-trained research participants at two different time points. No significant differences were found between any parameters. Shown are group means (± SD) for capacity, leak point pressure (LPP), maximum detrusor pressure (MDP) and contraction duration (CD).

Four different research participants were recruited for usual care assessments (at home in usual routine between measures–no training or any other study intervention) where two baselines were done at least three months apart (urodynamic measures and questionnaires). The research participants’ clinical characteristics are provided in Table 1 (Participant# NT where NT = non-trained). Note that although both males and females were being recruited for the usual care group, only males met that study’s inclusion criteria at the time of enrollment. Three of the four participants had indirect awareness (two above lesion level only) of need to empty bladder. There were no changes in sensation, bladder emptying times, incontinence episode frequency and cystometry parameters (see Fig 11) between the two study time-points. No pre-/post-usual care differences were found for either bowel (group average of 55 versus 60 minutes for defecation time) or sexual function (6.3 out of 10 points for desire and 2 out of 10 points for overall satisfaction for both pre- and post-usual care).

## Discussion

Improvements in multiple aspects of urogenital and bowel function, including bladder capacity, voiding efficiency, frequency of nocturia and urinary incontinence, time required for defecation and sexual desire underscores the power of LT for not only motor rehabilitation but for the benefit of multiple non-locomotor systems, including those involving autonomic functions. Although based on a small sample size, these pilot trial results suggest that an appropriate level of sensory information provided to the spinal cord, generated through task-specific stepping and/or loading, can positively benefit the neural circuitries controlling urogenital and bowel functions.

### LUT dysfunction

Bladder capacity at pre-training baseline, examined without taking any dosages of anti-cholinergic medication within 24 hours of testing, was on average substantially below the normal range for adults (300–400 ml; [86]), confirming the need to achieve a reasonable capacity to keep the number of catheterizations per day at a manageable number. In contrast, capacity post-training was on average nearly double (close to the low end of the normal range), although the number of daily catheterizations did not change (only change was at night–see discussion of nocturia below).

The initial objective was to determine the effects of LT on bladder function and was not geared toward altering the research participants’ adherence to their daily management program. Any management changes documented with the questionnaires were made on the research participants own volition and did not result from any instruction given by the research staff or study physician. Given the LT effect, one goal moving forward now that a significant increase in capacity was found would be to monitor and assist research participants under the supervision of a urologist on strategies to manage the reduction of their medication and/or the number of daily catheterizations. Implementation of quality of life measures during the process of altering participants’ bladder management program would be essential. However, before this can be attempted clinically, additional studies using a larger sample size are needed to establish how much, if any LT would be needed long-term to maintain these levels so these individuals would likely be able to reduce or even eliminate (for those with capacities above 300 ml) their bladder medication which has undesirable side effects. Although the usual care group of four participants (all male) addresses reproducibility of our outcome measures without undergoing any therapeutic intervention, future studies should also include a larger cohort of fully matched subjects that includes both males and females that reflect the SCI population, which is 80.6% male.

Another goal is to see potential additional benefits with a combinatorial approach that includes activity-based therapies. Current studies are underway with epidural stimulation alone and in combination with LT and/or stand training [87] to determine if capacity can improve even further. Sacral neuromodulation with implantation of the Interstim^®^ device (Medtronic Inc., Minneapolis, MN) has shown some positive effects in terms of increasing capacity with SCI individuals [88, 89]. One study using vibratory stimulation against the frenulum of the penis for ejaculation also showed a significant increase in bladder capacity (up to 3 minutes of stimulation every third day for four weeks) [90].

Although increasing capacity is vital for improving bladder management by decreasing the number of catheterizations and thus the incidence for UTI, it also is important to have a concomitant decrease in detrusor pressure during filling and during a void (detrusor leak point pressure), as pressures above 40 cmH_2_O puts the individual at higher risk for upper urinary tract deterioration over time [11, 91, 92]. As seen in Fig 2, pressures remained low during filling post-training despite the large increase in capacity. Also, leak point pressure was reduced post-training to an average pressure of 42.7 cmH_2_O, close to but not quite below the desired threshold value. Note that with LT, the participant (B23) having a suprapubic catheter (C5 level injury impacted manual dexterity and thus ability for catheterization), had a substantial decrease in leak point pressure despite having only a small incremental change in capacity. Continuous drainage of urine with an indwelling catheter results in a reduction in the size of the bladder and thus very low capacity.

Individuals with cervical and upper thoracic level lesions (majority of SCI population [93]) are at high risk for autonomic dysreflexia (AD). Bladder distention is one of the primary triggers of AD [83, 84]). Thus, it is important to note as illustrated in Fig 4 that the onset of AD with bladder filling during cystometry (> 20mmHg increase in systolic blood pressure—[83, 84]) post-training coincided with the change in capacity and not the pre-LT fill volume AD onset volume. The sensations above lesion that the research participants reported in the questionnaires (“awareness of need to empty the bladder”) are symptoms of AD (forehead pressure/headache; chills/piloerection; warm sensation/skin flushing) and appear to get used by SCI individuals to gauge the need to catheterize. The potential high frequency of this type of undocumented practice is a health concern and emphasizes the need for better therapeutic interventions.

Novel sensations below lesion post-training in one AIS A research participant (chills down both legs after undergoing 30 sessions of LT) suggests the existence and perhaps training-induced activation of dormant intact axons traversing the lesion. In another AIS A participant, perceived bladder sensations were reported pre-/post-training, which could reflect the existence/activation of extra-spinal sensory pathways. A recent fMRI study found significant activation of brain regions that receive inputs from the vagus nerve in 8 of 12 AIS A male and female individuals during bladder filling [94]. Data from several human and animal SCI studies support the possibility that the abdominal branches of the vagus supply the pelvic viscera below the left colic (splenic) flexure in SCI females (see [95, 96] and discussions in [97, 98]). Note that the AIS A research participant in the current study reporting in baseline questionnaires having experienced below level bladder sensations was female.

Four research participants reported at least one nightly awakening to catheterize (nocturia) at the pre-training baseline assessment. Only two of those four research participants reported nocturia at the post-training time-point. The fact that not all study participants reported initially waking at night to catheterize likely reflects restriction of fluids past a certain hour in the evening (another health concern) and not an absence of polyuria, the over-production of urine that occurs with SCI. Polyuria, examined in our SCI animal model, was shown to be prevalent even with mild contusion injuries [99] and was reduced in rats receiving 80 daily one hour but not daily 30 minute sessions of activity-based training [99, 100]. As reflected in the current results, the urologic improvements with LT in rats was significant but still not back to pre-injury levels, suggesting a combinatory approach may be needed. Current mechanisms under investigation as potential targets for polyuria include arginine vasopressin (AVP), a hormone secreted from the posterior pituitary which controls fluid homeostasis in the body [101]. Several studies have reported that following chronic SCI, AVP levels do not show the nightly increase that occurs in non-injured individuals [102, 103]. As a result, sleep is disrupted for both the individual and/or caregiver due to the increased need for catheterization. Polyuria may also lead to AD due to bladder over-distention and is associated with low morning blood pressure values and postural hypotension [104, 105]. Importantly, plasma and urine AVP levels have been shown to increase in able-bodied individuals receiving high intensity exercise [106–109]. Recent animal data from our laboratory show a significant decrease in serum AVP levels two weeks post-contusion at T9 [100]. In addition, examination of urine samples collected from the research participants reporting decreased symptoms of nocturia show a 95.8% and 64.5% increase in urinary AVP levels (AVP/Creatinine ratio) from pre-training baseline (using an enzyme-linked immunoassay; unpublished observations).

### Bowel dysfunction

Because of difficulties with elimination, large amounts of time are devoted to bowel care programs. Previous studies have found that toileting procedures may take up to 2–3 hours in SCI populations, with reduced efficacy of defecation and lack of independence in toileting [110]. During a food ingestion phase in one study that used a manometric catheter with four pressure transducers spaced approximately 10 cm apart, SCI individuals (n = 8) had substantially lower values in all colonic parameters measured (motility index, number of peristaltic waves, % activity, and mean amplitude of the wave motor pattern) relative to controls [111]. Pre-training data documented using the International Data Set [75] for the present study research participants showing a mean toileting time of 57.9 ± 6.9 minutes (n = 8) to complete bowel care is consistent with the limited existing published data. Prolonged transit time has been found to be an important part of the mechanism underlying constipation in SCI individuals [112, 113]. A significant reduction in time (35.7 ± 26.2 minutes) was found with activity-based training in the current study which could reflect a change in motility, a disturbance hypothesized to result from the loss of descending modulation of the sympathetic supply [6] and thus an imbalance between the parasympathetic and sympathetic nervous systems. A reduction in time spent in bowel care has previously been shown in a multicenter trial using an implantable neuroprosthesis device for sacral nerve stimulation [114]. Studies with epidural stimulation alone and in combination with LT and/or stand training are now in progress to determine potential benefits on bowel timing as well as motility (in addition to bladder function).

### Sexual dysfunction

Modest ongoing engagement in sexual activity (either alone or with a partner) around the time of the study limited questionnaire data to element domains regarding primarily overall satisfaction and desire. Low levels of engagement are associated with multiple factors, such as years’ post-injury (in terms of likelihood of having a relationship–greater with time), age, spasms, pain and the presence/absence of AD during sexual activity [115]. Overall satisfaction scores above the minimum of “very dissatisfied” in the current study coincided with the presence/absence of sexual activity at the time of data collection. These variable satisfaction scores are consistent with the strong existing association between sexual function and quality of life [116–118], which is not surprising given web-based survey results indicating that the primary reason for pursuing sexual activity is need for intimacy and not sexual need or fertility [115].

The baseline sexual desire values obtained for the research participants in the present study were consistent with surveys that have documented high levels of sexual interest, particularly in comparison to other domains such as satisfaction [118, 119]. These findings underscore the need for continuous intervention by health professionals for guidance on treating sexual dysfunction in the SCI population. Sexual desire was found to be significantly higher post-training, which likely relates to well-known exercise effects on libido. For example, physical resistance training significantly enhanced FSFI sexual desire domain scores in women with polycystic ovary syndrome [120] and physical activity significantly increased multiple IIEF domains including desire in men receiving phosphodiesterase type 5 inhibitors (PDE5i) for erectile dysfunction versus PDE5i alone [121].

In male SCI participants, erectile function pre-training baseline data from the EHGS and IIEF are consistent with intact sacral circuitries that allow for reflexogenic erections but as revealed in previous studies are insufficient for sustainability and lack enough rigidity for penetration [122–124]. A lack of a training effect in the present study is likely a function of the limitations of the outcome measure scoring, as multiple participants reported that they had noticed a change in rigidity and/or longer lasting erectile episodes. Pressure recordings [124] in future studies would provide a more quantifiable less subjective outcome measure of penile tumescence.

Questionnaire outcomes with respect to a lack of ejaculation (either pre- or post-training) are consistent with severe impairment of critical descending circuitries for supra-spinal projections that mediate the somatic-parasympathetic coordination that is necessary for expulsion of semen [8]. Most of the male participants reported anecdotally seminal fluid in the bladder after sexual activity, reflecting poor bladder neck muscular control (normally contracted to prevent retrograde ejaculation into the bladder) [125, 126].

## Supporting information

S1 File Study protocol. Detailed information on NICHHD-funded study protocol, including significance, innovation and approach.
(PDF)

S2 File Institutional Review Board (IRB) approval letter. University of Louisville IRB approval document for the study “Effects of activity dependent plasticity on recovery of bladder and sexual function after human spinal cord injury”.
(PDF)

S1 Table Transparent Reporting of Evaluations with Nonrandomized Designs (TREND) statement checklist. A 22-item checklist used to guide standardized reporting of nonrandomized controlled trials.
(PDF)
