# Supplementary material for: Improvements in bladder, bowel and sexual outcomes following task-specific locomotor training in human spinal cord injury
Source: PLoS One. 2018 Jan 31;13(1):e0190998. doi: 10.1371/journal.pone.0190998 (PMC5791974; doi:10.1371/journal.pone.0190998)
Supplement: S1 File — Detailed information on NICHHD-funded study protocol, including significance, innovation and approach. (PDF) [file pone.0190998.s001.pdf]

B. SIGNIFICANCE

Approximately 1,275,000 people in the U.S. live with paralysis from spinal cord injury (SCI) [1]. Disabling SCI sequelae include impairments of locomotor, sensory and autonomic functions [2, 3]. The U.S. could save an estimated $400 billion on direct and indirect lifetime costs if we can develop therapies to treat SCI. Also, the cost in emotional stress and well-being to the individual and family is demanding. Bladder and sexual dysfunction consistently ranks as one of the top disorders drastically affecting quality of life after SCI [2, 3]. Urological complications are responsible for most clinical conditions and hospital readmissions [4], and individuals with SCI are eleven times more likely to die from diseases of the urinary system than non-injured individuals. Locomotor Training (LT) has shown a range of benefits on health and function in both human [5-17] and animal [18, 19] models. Improved bladder and sexual function has been reported by patients undergoing LT even years after injury; however this phenomenon has not been studied. The mechanistic insights of how LT affects not just walking recovery but urological and sexual function not only will lead to imminent treatments for those suffering with SCI but may influence the treatment of other neurological disorders such as multiple sclerosis, Parkinson's, and stroke, and stimulate investigations on other systems such as cardiovascular, pulmonary and bowel dysfunctions.

1. Urological Dysfunction and SCI. Deficits in urological function after SCI manifest as: detrusor hyperreflexia (bladder contractions at low volumes, causing incontinence and smooth muscle hypertrophy), detrusor-sphincter dyssynergia (uncoordinated bladder and external urethral sphincter contractions, causing inefficient emptying and smooth muscle hypertrophy), decreased compliance (unable to store urine under appropriately low pressures) and loss of continence requiring lifelong management, maintenance, and health care visits [20]. Current therapies to improve efficiency of bladder voiding, management and continence after SCI include catheterization, pharmacologic and surgical interventions, functional electrical stimulation, and urethral stents [21, 22]. Catheterization can induce scarring, stricture formation, cystitis and frequent urinary tract infections due to introduction of bacteria into the urethra [23]. Pharmacological agents alter internal urethral sphincter tone (α blockers) or relax the detrusor muscle (anticholinergics increase bladder compliance and decrease
pressure) but do not treat voluntary voiding. These drugs have side effects like dry mouth and constipation further complicating the issue of fluid restriction to control timely urine evacuation [21, 24]. Surgeries to reduce high bladder pressures and chronic urinary incontinence include urinary diversion and lower urinary tract reconstruction [22, 25, 26]. Surgery to implant neural control devices to artificially influence voiding circuitry often require ablation of intact neural tracts. There is a critical need for a successful treatment that restores normal lower urinary tract function as the compensatory strategies continue to diminish the capacity of the bladder, require life-long maintenance, have deleterious side effects and lead to recurring illness .

2. Locomotor Circuitry and Bladder Function. The interaction of lower limb musculature with the bladder and its sphincter has been observed sporadically over the years, as far back as 1933, in both humans [27, 28] and animals [29, 30]. Flexor and extensor reflexes can be modulated by the state of bladder filling and voiding in both normal patients and those with CNS damage [28]. In patients with spasticity, the general pattern is that detrusor contractions precede limb flexor spasms [31]. Our experience with patients undergoing LT reveals two important observations: 1) catheterization is required before each session because a full bladder will inhibit stepping; and
2) an individual who received LT in combination with epidural stimulation of the lumbosacral region regained the ability to routinely void without catheterization. These observations suggest an interaction of the locomotor and bladder circuitry that is emphasized after SCI. Afferent input from the bladder and/or the external urethral sphincter affects the limb musculature and repetitive activation of the locomotor circuitry seems to affect bladder function. We propose that this vesicosomatic relationship can be influenced by LT to enhance bladder integrity and function and improve the health/quality of life for those suffering from SCI.

Recent discoveries in humans related to activity-dependent plasticity have led to a widely implemented activity-based (generates neuromuscular activation below the level of injury) rehabilitation intervention, LT,

for those with incomplete SCI [14, 32-35]. The therapeutic intervention is usually implemented in those with incomplete injuries, even though the mechanistic studies have been done in clinically complete SCI, because while LT optimizes the spinal circuitry, remaining residual supraspinal inputs may be required to sufficiently excite these networks for successful walking [36]. Generation of locomotion by the interaction of afferent input with central pattern generating networks has been shown in spinally transected animals [18, 37-46] and several of these properties exist in the functionally isolated human spinal cord [10, 11, 47-52]. Motor patterns observed during

stepping in individuals with clinically complete SCI [11, 39, 53, 54] are driven by sensory information available to the spinal cord interneuronal networks [47, 51, 55, 56]. Multiple sensory inputs from the periphery during locomotion, particularly limb loading [57] and stepping rate [58] provide information to these networks to improve stepping [53, 55, 59-61].

One individual with a clinically motor complete SCI did not produce motor activity in the legs even after 170 sessions of LT. However, in the presence of epidural stimulation, he was able to stand independently and voluntarily move his legs indicating that the spinal circuitry requires a sufficient level of excitability to execute motor tasks. Intense, repetitive LT with sufficient spinal network excitability (via residual supraspinal input or epidural stimulation) conceivably induces a net improvement in functional reorganization of the neural circuitry in response to afferent feedback and supraspinal commands and/or epidural stimulation [62-65]. Improving these synaptic inputs might therefore also lead to adaptive changes to other systems such as those controlling the bladder and genitalia since much of the motor and autonomic output of the spinal cord is driven in large part by afferent input and local or propriospinal circuitry emphasized after SCI conditions [51, 66-69].

3. Sexual Dysfunction and SCI In men with SCI, the degree of sexual dysfunction depends on level of the lesion. For our research, only injuries cranial to T10 are considered as the spinal reflex arcs for erection and ejaculation are considered to be left intact, with only the removal of supraspinal input [70]. Most individuals with SCI's above T10 demonstrate reflexogenic erections of varying degrees in response to very slight stimulation of the penis [71, 72]. Though erections are easily initiated, they are not easily sustained [73] which has been proposed to be a result of altered penile sensitivity [74]. In 95% of SCI men with lesions cranial to T10, normal ejaculation is severely impaired or impossible [72, 75] despite the intact spinal reflex arc, suggesting the ejaculatory reflex circuitry is more dependent on supraspinal control than is the erection circuitry. The ejaculatory dysfunction may also relate to the decrease in penile sensation. Many SCI individuals who do not respond to normal tactile stimulation of the penis may ejaculate to intense vibratory stimulation of the ventral penile midline [76] suggesting that massive recruitment of all low and high threshold penile mechnoreceptive afferent neurons can provide enough input to the spinal ejaculatory circuit. In addition to erectile dysfunction and ejaculatory failure, abnormal sperm motility and viability as early as two weeks post-SCI contribute to neurogenic reproductive dysfunction post-SCI [77].

4. Neurotrophins. Exercise and neuromuscular activity are highly influential on expression of neurotrophins (such as BDNF and NT-3 in lumbar spinal cord and soleus muscle - [78]) known to modulate cellular and synaptic function in the adult [79-82]. Among various strategies, step training after contusive SCI in animal studies promotes the greatest changes in neurotrophin production and functional recovery (such as the normalization of BDNF levels in the lumbar spinal cord and soleus muscle - [83]). Other forms of exercise attempted in the contused rat model, such as swim training, only had a transient effect, and stand training had no effect on neurotrophins and functional recovery [83].

Transplantation of NT-3/BDNF expressing fibroblasts into the injury site after contusive SCI has been reported to increase bladder and locomotor function [84] and viral-vector-mediated delivery of NT-3 via i.m. injection after SCI promotes locomotor recovery and electrophysiological changes in motoneurons akin to training [80]. Combined step training and neurotrophin therapy results in greater locomotor gains than either alone [85]. Although increased levels of NT-3/BDNF appear to be adaptive/beneficial when expressed in the spinal cord or muscles (at least for locomotor functions), increased NGF in the bladder and corresponding spinal segments plays a significant role in dysfunction. Upregulation of neurotrophic factors post-SCI [86] is responsible for the re-emergence of the spinal voiding reflex within 2 weeks of injury [87], but chronic changes in NGF appear to be responsible for bladder afferent hypersensitivity, hypertrophy, and sprouting of axons [88], all of which lead to bladder over-activity. NGF and BDNF have been implicated as key factors in a variety of bladder dysfunctions [89]. NGF delivery (intrathecal infusion at L6-S1 spinal level in adult rats) causes bladder DRG afferents to become hyper-excited and results in detrusor hyperreflexia [90], while NGF removal, via antibody treatment (intrathecal infusion at L6-S1 spinal level in adult rats), has been shown to relieve detrusor hyperreflexia and detrusor-sphincter dyssynergia [91, 92]. Sequestering BDNF (daily tail vein administration of TrkB-Ig_2, which specifically binds BDNF and neutralizes it) has been shown to improve bladder function in a chronic cystitis model [93]. Exercise therapies also influence neurotrophin expression in visceral target organs, not just skeletal muscle [94, 95]. We therefore hypothesize that LT and epidural stimulation will influence neurotrophin levels within the spinal cord and periphery, leading to enhanced locomotor and visceral function.

C. INNOVATION.

- This proposal focuses on the effects of activity dependent plasticity induced by locomotor training following chronic SCI on non-locomotor systems, those involved with bladder and sexual function.

- Bladder and sexual dysfunction after SCI is rarely studied in experimental animals, yet is overwhelmingly the most significant concern for those suffering from SCI and urological complications results in significant morbidity and mortality. In addition, small improvements in bladder and sexual function can have a tremendous impact on these individuals' continual health and quality of life.

- This proposal involves the collaboration of scientists with extensive experience in animal and human SCI models. In our currently funded animal study, rats undergoing step training are also being compared to a SCI group receiving the same intensity of exercise without weight bearing by stepping on only their forelimbs (equivalent to the arm crank control group proposed here), as well as to an uninjured group. These ongoing parallel studies in animals will provide further insights to underlying mechanisms that can also be tested in the human model.

- A novel multi-disciplinary combination of outcome measures including EMG recordings, stepping kinematic measures, urodynamics, International Index of Erectile Function, and molecular assessments of bladder tissue will also be conducted in this study. Histological, biochemical, and molecular assessments of spinal cord and dorsal root ganglion tissue are in the process of being conducted in rats to identify specific mechanisms not testable in humans.

- The current proposed study will increase our understanding of human lumbosacral spinal networks and guide the use of innovative therapeutic strategies that would be immediately available to not only improve the motor output during standing and walking but ameliorate bladder and sexual dysfunction and thus improve quality of life in individuals after SCI.

APPROACH

1. General Design. Two cohorts of individuals who sustained a SCI will be studied. The first cohort includes 3 groups of 10 subjects (30 in total over a 5 year period) evaluated before and after receiving either a standardized locomotor training (LT) program, a standardized stand only program, or a standardized arm crank exercise program that is provided clinically at Frazier Rehab Institute within the NeuroRecovery Network (NRN) [11] (***Specific Aims 1 and 2***). The second cohort includes 10 subjects evaluated before and after receiving epidural stimulation in combination with stand training then LT that is being provided by our research team at the University of Louisville Human Locomotion Research Center (n=10, ***Specific Aim 3***). The individuals in the second cohort will receive epidural stimulation (See Appendix A for details) in combination with stand training as participants in IRB#07.0066 or IRB#13.0625. For Aims 1 and 2 studies, individuals who sustained a SCI (n=30) will be randomized into three groups that receive either step training (n=10) or stand training (n=10) or arm crank exercise (n=10). Blocked randomization will be employed
to preserve treatment balance.

Each individual <u>will serve as their own control</u> reducing the variability among individuals related to the injury itself, time since injury, medications taken, therapies received, differences in degree of sexual dysfunction, and many other factors that cannot be controlled in the human experience. The inclusion criteria for both cohort research participants are: 1) stable medical condition without cardiopulmonary disease or dysautonomia that would contraindicate LT; 2) no painful musculoskeletal dysfunction, unhealed fracture, contracture, pressure sore or urinary tract infection that might interfere with training; 3) no clinically significant depression or ongoing drug abuse; 4) clear indications that the period of spinal shock is concluded determined by presence of muscle tone, deep tendon reflexes or muscle spasms and discharged from standard inpatient rehabilitation; 5) no current anti-spasticity medication regimen; 6) non-progressive SCI above T10; 6) bladder and sexual dysfunction as a result of SCI. Urodynamic parameters, erectile dysfunction evaluations, urine biomarker and biopsy sample retrieval, and EMG activity assessments will be conducted as outlined in Figure 1.

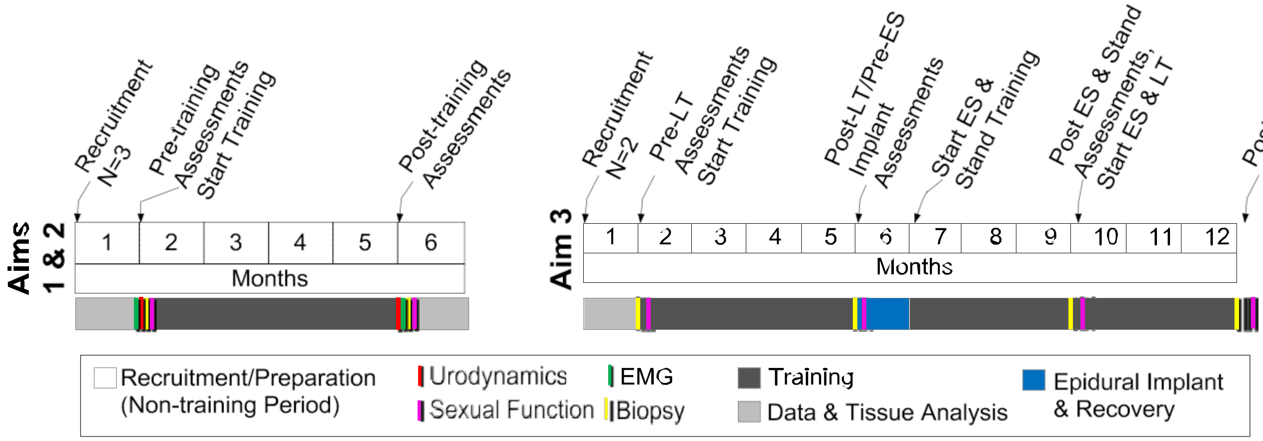

Figure 1. Timeline for Aims 1 and 2 (left) and Aim 3 (right). For Aims 1 and 2 research participants with incomplete SCI (American Spinal Injury Association Impairment Scale (AIS) B, C or D [96-98]), urodynamic/erectile function assessments and biopsies will be conducted prior to enrollment in the standardized NRN LT program and after 80 sessions of the intervention. Training will be done in groups of three (one LT, one stand training, one arm crank training). For Aim 3, urodynamic/erectile function assessments and EMG activity will be conducted on individuals with severe SCI (those who are motor complete and sensory complete [AIS A] or sensory incomplete [AIS B]) (i) before LT, (ii) after 80 sessions of LT prior to implantation, (iii) after 80 sessions of stand training in combination with epidural stimulation (ES), and (iv) after 80 sessions of LT in combination with epidural stimulation (n=2 per year for a total of 10 over 5 years).

2. Approach for Specific Aims.

a. Specific Aim 1: *To determine the effects of weight-bearing task-specific training for locomotion (stepping on a treadmill) after traumatic incomplete and complete SCI in humans on a) urodynamic parameters, b) interactions between lower limb and urinary bladder circuitries, and c) bladder neurotrophin levels. Weight-bearing (stand-only) and non-weight-bearing exercise (arm crank) will serve as controls.*

SCI research participants are expected to exhibit marked urological deficits (e.g., detrusor hyper-reflexia with detrusor-external sphincter dysynergia). An example showing a baseline cystometrogram (to demonstrate feasibility) from a 32 year old male, obtained 4 yrs. 3 mo. post-SCI (C7 level; AIS B), is provided in Figure 2.

We hypothesize that individuals with severe SCI after LT will have significant improvements in continence and voiding ability, as evidenced by an increase in bladder capacity, lower detrusor pressures and voiding pressures, the ability to resume normal voiding at least partially without catheterization, and lower post-void residual volumes.

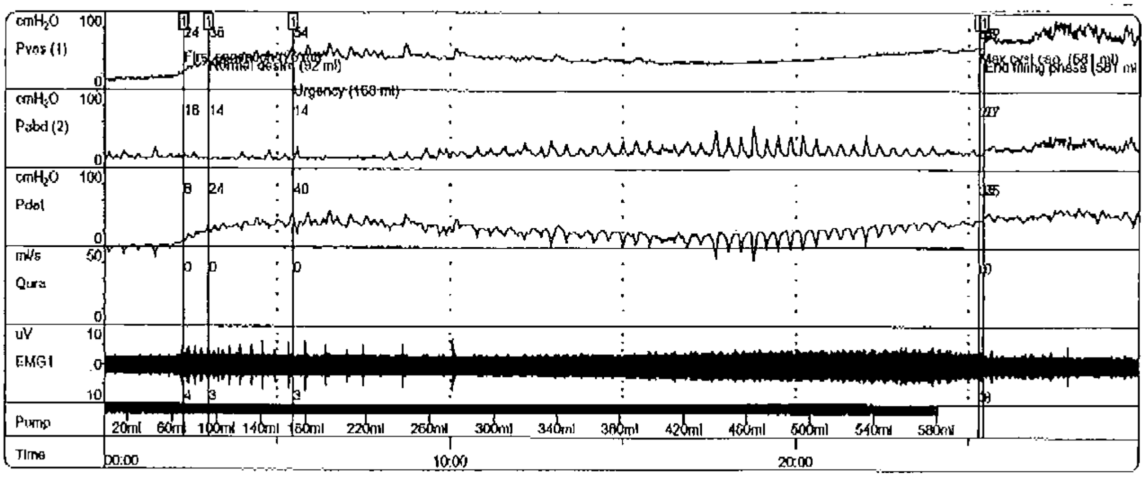

Figure 2. At the beginning of the filling curve at around 60 ml, the subject had an uninhibited bladder contraction that reached 40 cm H_2O of pressure, demonstrating bladder overactivity and low compliance. The bladder was still able to be filled up to 580 ml, as after the initial bladder contraction the pressure decreased

only to rise again to 40 cmH_2O. After the end of the filling phase, the subject was asked to void, but was unable to achieve any flow to what appeared to be detrusor sphincter dyssynergia type 3 as the sphincter EMG activity remained high during the entire voiding phase.

Cystometry measures obtained from a T5 AIS Grade A subject (two years post-SCI) during preliminary studies of LT effects on bladder function indicates an increase in compliance and doubling of volume at first uninhibited bladder contraction after 80 daily sessions of LT (Figure 3). The recording in Figure 3 provides an example of how cystometry *(see details below in General Methods)* measurements of resting pressures, bladder capacity, and voiding volumes will be quantified. Lower resting pressures reflect the ability of the bladder to store more urine (indicative of a change in compliance). For the current proposed clinical study, we will also measure changes in voiding frequency over time using established documented voiding diaries. Therefore, improvements in a variety of specific quantifiable parameters related to bladder function are expected to contribute to better continence as well as improved voiding efficiency revealed by a decrease in residual volume and voiding frequency, an outcome that would likely lead to improved urological health and quality of life for those suffering from SCI.

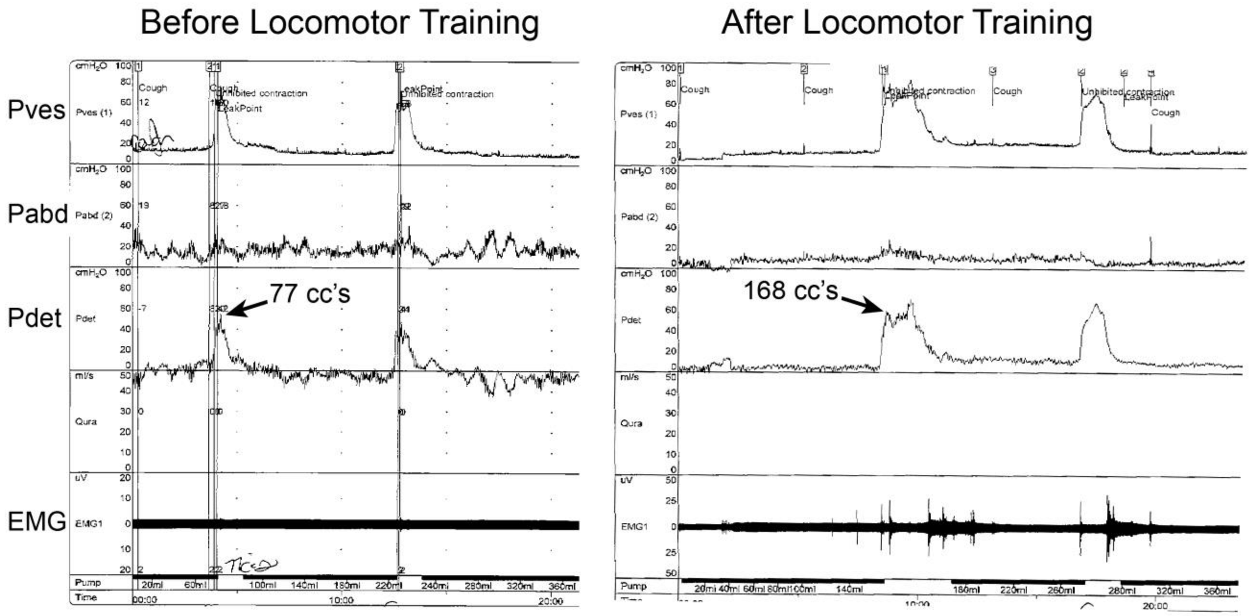

Figure 3. Example of cystometry recordings from a 29 year old male AIS Grade A (motor and sensory complete SCI) two years after injury (T5 neuro level). Improvement of compliance and doubling of the volume at the time of his first uninhibited bladder contraction was noted after 80 sessions of LT. Note that this individual's bladder medications were discontinued 24 hours prior to each of the cystometry sessions. In addition, this individual reported having chills through both legs when his bladder was full after, but not before LT (he said he started noticing the sensation after several weeks of training). Note that he is sensory complete as determined by AIS standards and the fact that he could tell when his was full (and thus the need to catheterize) was a huge benefit for him (we have found similar reports for all four subjects that have been studied to date in these preliminary studies, although the location and type of sensation has varied – see Specific Aim 3 below).

Data from our animal studies (Figure 4 below; manuscript in preparation) shows that following 80 step training sessions the ability of SCI rats to empty the bladder increased. The mean voiding efficiency (percent volume voided/volume infused) of the trained group was significantly greater than non-trained (Figure 4a, trained 39.78% ± 17.72; non-trained 22.29% ± 11.02, p=.042). This was accompanied by a significant increase in the maximum amplitude of bladder contraction (MAC, pressure in mm Hg) and a significantly increased intercontraction interval (ICI, time in seconds) (Figure 4, MAC p=.043, trained 6.54±4.75 non-trained 2.56±1.39; ICI p=.018, trained 25.40±12.98 non-trained 12.25±3.77). Contraction time (trained 25.23±15.35 sec, non-trained 16.03±3.67 sec), resting pressure (trained 16.10±5.52 mmHg, non-trained 17.80±2.46 mmHg), and bladder weight (trained 0.36±0.11 g non-trained 0.40±0.10 g) demonstrated no differences between groups. However, bladder weight (grams) significantly correlated with voiding efficiency in only the

trained group ($p=.004$, $r= -.946$, $r^2=.895$, $n=6$). Bladder hypertrophy post-SCI can result from detrusor sphincter dyssynergia in a manner similar to bladder outlet obstruction which is reversible with outlet relief [99, 100]. The sphincter must be relaxed during a bladder contraction to allow emptying. We can infer that the sphincter of the trained rats was in partial coordination to allow for the flow of urine, an increase in voiding efficiency, and the significant relationship between bladder weight and voiding efficiency for trained rats.

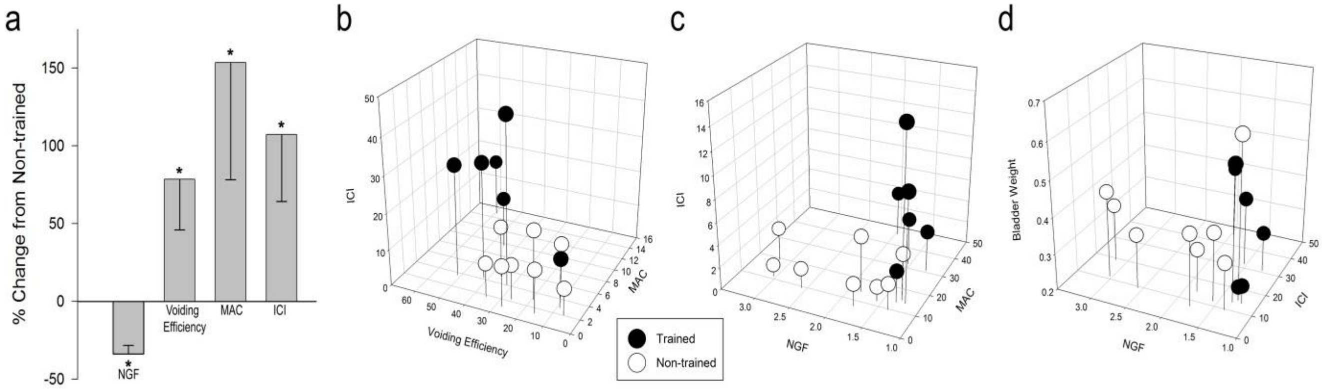

Figure 4. Bladder NGF mRNA and Cystometry. a) Percent change from the non-trained mean for NGF, voiding efficiency (percent volume voided per volume saline infused), maximal amplitude of contraction, and intercontraction interval. Mean NGF significantly decreased and mean voiding efficiency, intercontraction interval (seconds), and maximal amplitude of contraction (mmHg) significantly increased with training. b) 3D relationship between cystometry measures. c & d) 3D relationship between NGF and cystometry measures. Significant group differences were detected by group using multiple regression analysis (MAC and ICI were good predictors of voiding efficiency with significant group differences ($R^2=.870$, $p<.05$, $N=14$)). Therefore, training modified the relationship of voiding efficiency, MAC, and ICI.

However, it is not clear whether step training induced bladder recovery is due to activation and plasticity in lumbosacral circuitry (bladder and locomotion). To assess potential underlying mechanisms in the current proposed study, bladder neurotrophin levels are also being evaluated after SCI. We expect that LT and epidural stimulation will affect the levels of bladder neurotrophins in a manner that enhances functional recovery. We hypothesize that SCI will induce an increase in NGF and BDNF in urine and biopsied bladder tissue and this will be reversed with LT but not with arm crank or stand training. Note that a study in rats using different exercise paradigms (treadmill training, swim training, stand training) found significant improvements on central pain and normalization of lumbar spinal and soleus muscle BDNF levels for the LT group only [83]. Bladder tissue from our preliminary study in rats was analyzed for NGF mRNA using real-time PCR (Figure 4). Multiple regression analysis of selected cystometry parameters with NGF revealed that two of the cystometry measures and bladder weight were good predictors of NGF with significant group differences. ICI and MAC were significant predictors of NGF ($R^2=.638$, $p<.05$, $N=14$). ICI and bladder weight were significant predictors of NGF ($R^2=.689$, $p=.004$, $N=14$). Therefore, training modified the relationship of NGF to ICI, MAC, and bladder weight. These relationships are demonstrated with 3D graphs in Figure 4b-d.

Lower NGF levels significantly correlates with lower residual volume, indicating higher voiding efficiency and reduces the potential for recurring urinary tract infections. The relationship between NGF and residual volume is apparent only in association with LT, which may be due to altered circuitry that controls micturition after SCI. We chose to examine NGF first because previous reports have shown that it is up-regulated in the bladder following SCI and it is highly associated with bladder dysfunction. NGF delivery causes bladder afferents to become hyper-excited and results in detrusor hyperreflexia [90], while NGF removal, via antibody treatment, relieves detrusor hyperreflexia and detrusor-sphincter dyssynergia [91, 92]. Treatment that lowered bladder BDNF levels reduced over-activity in a cystitis rodent model [93] and thus is an additional neurotrophin that we will target. In our current animal studies, to broaden potential targets, we are conducting a focused neurotrophin qPCR array using bladder tissues from trained versus non-trained male rats. The results could be used to identify one or two additional targets to examine in the human tissues we obtain for the current study. Thus, our new rat data (Figure 4) indicates for the first time that the post-SCI bladder NGF levels and bladder function can be affected in tandem by LT. This finding would indicate the need to maintain some daily form of

training, perhaps in combination with a drug therapy (e.g., anti-NGF). These experiments will provide new knowledge of the role of training on the neurotrophin regulation in humans after SCI.

Similar results are anticipated for urine biomarkers for NGF and BDNF, an area of investigation that has received a lot of recent attention for overactive bladder. Several studies have been published indicating elevated urinary levels of NGF and BDNF (but not GDNF) concentrations (measured by ELISA and normalized to creatinine) in patients with overactive bladder relative to healthy controls [89, 101-104]. These levels have been shown to decrease back to normal levels with a variety of treatments, including antimuscarinic therapy [101, 105] and a 3 month lifestyle intervention (bladder training - limited and select fluid intake) [101]. As with overactive bladder, urinary biomarkers may be used for the evaluation of bladder dysfunction in SCI individuals. In addition, we may find a strong correlation with the neurotrophin levels in the biopsy samples (urinary biomarkers would make it much easier to monitor the effects of our therapeutic interventions).

We also hypothesize that after SCI, bladder filling at rest (tested while doing cystometry) will result in co-activation of lower limb flexors and extensors which will be significantly reduced after LT but not arm crank or stand training. We hypothesize that the locomotor activity generated during stepping influences the bladder circuitry and improvements in bladder function will correlate to improvements in EMG activity during stepping (measured as part of a study focused exclusively on locomotor circuitry) and reduce the aberrant activity during bladder filling. We will record EMG activity (*see details in Methods below*) of the lower leg muscles during the cystometric testing and during stepping on a treadmill (bladder is emptied prior to each 60 minute session, so it will not be fully distended at any point during training). Specifically, we expect that pre-training, increased tibialis anterior EMG activity (hyperactive flexion reflex) and co-contraction of extensor and flexors will occur in response to the bladder contractions. A fully distended bladder (filling during cystmetry session) is expected to trigger spasticity or clonus. Such a finding would explain the observed need for the bladders of human SCI individuals to be emptied immediately prior to rehabilitative step training on a treadmill. A full bladder may inhibit training by increasing EMG activity within flexors, thereby causing clonus or spasticity. We propose that this functional reorganization occurs with the LT intervention corresponding to improvements in EMG activity during stepping. These experiments will further our knowledge about vesicosomatic reflexes and understanding about how visceral organs may affect other systems, challenging our view of the locomotor system and the sources of sensory inputs to the central pattern generator. An example showing the benefits of LT on EMG activity of the lower leg muscles is provided in Figure 5 from both our human and animal study.

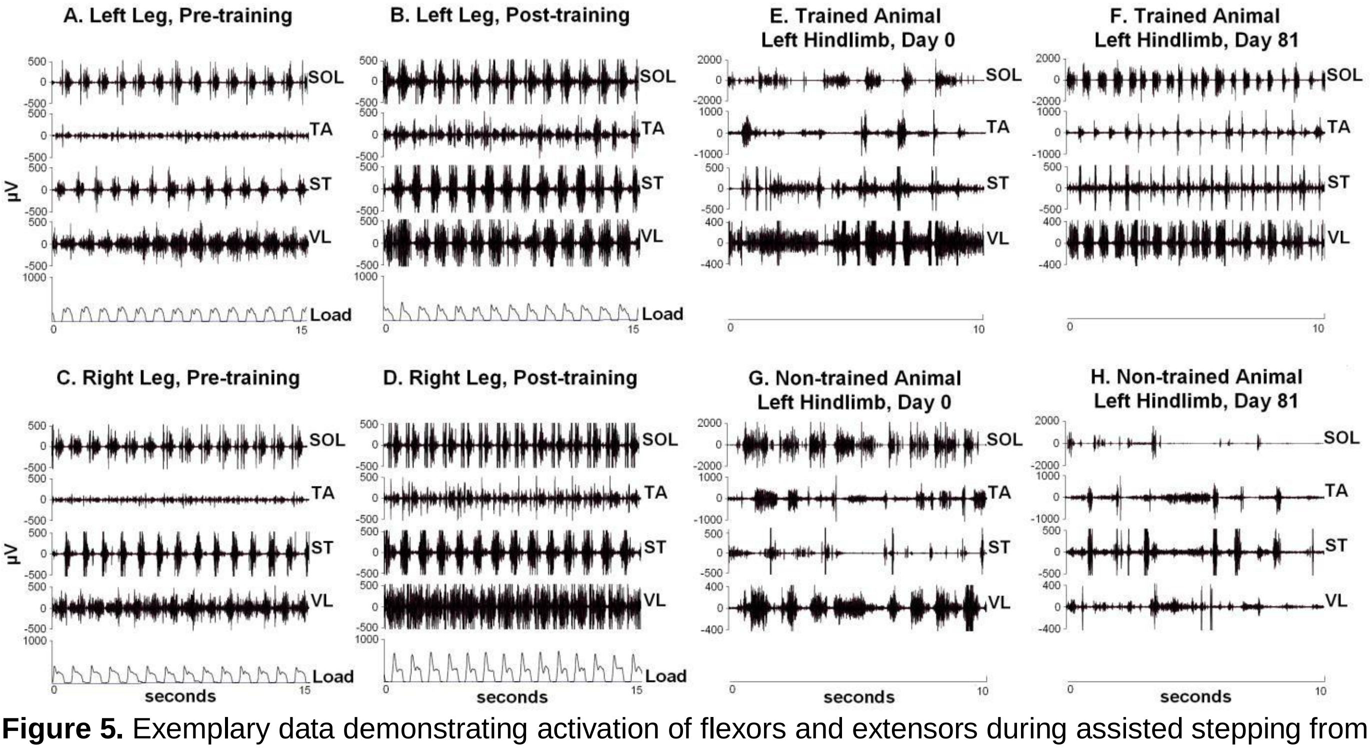

Figure 5. Exemplary data demonstrating activation of flexors and extensors during assisted stepping from an individual with a clinically incomplete SCI (Panels A-D) and unassisted stepping from a trained and non-trained adult rat (E-H). EMG activity (µV) from the soleus (SOL), tibialis anterior (TA), semitendinosus (ST), and vastus

lateralis (VL) from both left and right legs of a human pre- (A & C) and post-training (B & D) and from the left hindlimb of a trained and non-trained animal at Day 0 (E & G) and Day 81 (F & H).

b. Specific Aim 2: *To determine the effects of weight-bearing task-specific training for locomotion (stepping on a treadmill) after traumatic incomplete and complete SCI in humans on erectile function (stand-only and arm crank once again will serve as controls). Subjects will be the same as for Aim 1.*

SCI research participants are expected to exhibit marked deficits in sexual function (erection; ejaculation) and thus low levels of sexual satisfaction upon entering the study [106, 107]. Many are likely to be using drugs or sexual aids to enhance function. It is hypothesized that LT but not arm crank or stand training will result in a significant improvement in erectile function and sexual satisfaction as evidenced by a significant score increase in the International Index of Erectile Function (IIEF), a grade increase on the Erectile Hardness Grading Scale, and a significant reduction in the need to enhance erectile function with medications and/or aids. Note that the IIEF is the "gold standard" for evaluation of erectile function in clinical trials [108, 109] and is the preferred instrument for measurement in the SCI population [110]. Also, the Erection Hardness Grading Scale is often used along with the IIEF [111, 112]. Importantly, studies show that scores obtained with subjective grading of erectile hardness by patients is positively associated with psychosocial outcome measures (emotional well-being and sexual satisfaction) [112]. Preliminary assessments were obtained for one study subject before and after undergoing epidural stimulation plus LT (in addition to the many anecdotal reports from subjects undergoing LT at several rehabilitation centers that are part of the Neural Recovery Network). These preliminary findings are provided under Specific Aim 3 (section c below).

c. Specific Aim 3: *To assess the added effect of epidural stimulation with standing and LT on urodynamic parameters, bladder urinary neurotrophin levels and sexual function after severe complete SCI.*

It is possible that for individuals with motor complete SCI (AIS A, B) who generate very minimal levels of EMG activity during stepping, significant bladder recovery will not occur, as does not occur for standing and stepping, even after intensive LT. We can further test the hypothesis of activation of the spinal circuitry as a mechanism for improved bladder function by also studying a cohort of individuals who first undergo LT and then receive an epidural implant for the intent of generating standing and stepping (Hypotheses 3A – 3D). Dr. Harkema is the investigator of a NBIB funded study that focuses on the evolution of standing and stepping in humans using epidural stimulation combined with LT [113]. We can leverage these already funded and ongoing studies to further our knowledge of activating spinal circuitry in the recovery of physiological functions, including bladder and sexual function. We will complete the same assessments as in Aims 1 and 2 on subjects with motor complete SCI already enrolled in this study. These experiments will provide information on whether in the most severely injured individuals a combination of epidural stimulation and LT will be sufficient to evoke bladder changes. An example showing improved bladder function from the first study participant to have urodynamic assessments before and after epidural plus LT (recently completed) is presented in Figure 6.

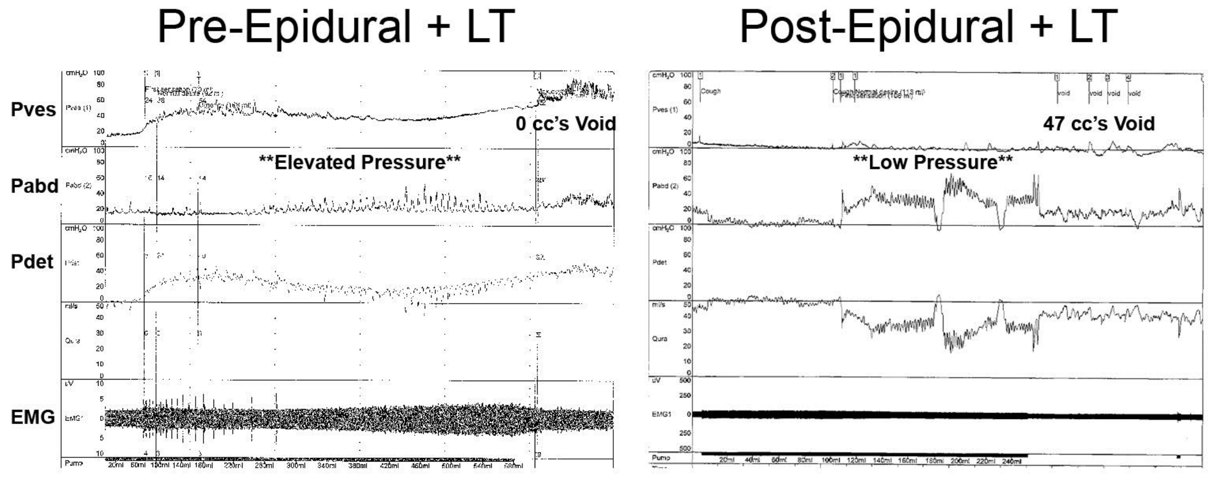

Figure 6. Cystometry recordings from a 32 year old male with a C7 neuro level SCI, AIS Grade B (motor complete, sensory incomplete; full pre- recording shown in Figure 2). Note the reduced pressure during filling and some ability to voluntarily void post-epidural plus LT. Urgency threshold was at 160 cc's pre-epidural+LT and 260 cc's post-epidural + LT. Note that during the voiding phase of the post-epidural + LT assessment, the

subject commented (i) that it was cold in the room, and (ii) there were lots of people around. Perhaps the void would have been more in a different environment. Asked if he tried to void on his own at home, the subject stated that he did not try as he did not want to risk getting a UTi.

In terms of sexual function, the same outcome measures will be done as per Aim 2. An assessment was done on a male subject (AIS Grade A; neuro level T4) receiving epidural stimulation + LT. Prior to enrollment in the study, the subject reported being a Grade 2 (increase in size and slight increase in hardness, but insufficient for sexual intercourse) and used the maximum dosage of Viagra (100 mg) 100% of the time to achieve a Grade 4 (fully hard [rigid] erection) on the Erection Hardness Grading Scale. After completion of the study and returning for a six month follow-up (had access to a trainer at home and did one hour sessions of stand training 5 days a week), the subject reported a Grade 4 score, and only needed to use a low dose of Viagra (25 mg) less than 50% of the time. He reported through interview that Viagra was needed on long work days when he did not have time or energy to do his daily training. He also reported that he noticed the changes in erectile function after about 3 to 4 weeks of epidural plus LT training during the study.

Note that all the preliminary data reported above comes from three of the four subjects to date. The first subject, whose locomotor outcome data was published in 2011 in Lancet [114], was trained prior to the start of collecting urodynamic data for bladder function. Also, Figure 3 (pre- and post-training example) is from the current study participant who just recently received an epidural implant. Although bladder function data is provided above for one subject (AIS B, Figures 2 and 6) and sexual function data for the other (AIS A described in previous paragraph), both participants had improvements in bladder and sexual function. The C7 AIS B participant, for example, had the ability to ejaculate but experienced head thumping during it (i.e., autonomic dysreflexia). The subject reported that the head thumping went away about one month into training plus epidural stimulation. The first study subject, a T2 AIS B, went from a score of 12 (mild to moderate erectile dysfunction) on the Sexual Health Inventory for Men (SHIM; short form of the IIEF) to a score of 24 (normal erectile function), with satisfaction rating for attempted sexual intercourse increasing from “a few times” to “many times” before versus after training plus epidural stimulation. All four subjects report increased awareness of bladder fullness after just LT, including chills down the legs (per description in Figure 3 legend), a sense of pressure below the umbilicus, and a sense of the penis retracting. The C7 AIS B subject, for example, would get a tingling sensation in his face and the top of his head (both sides) when his bladder is full (prior to enrollment in the study). After training, in addition to these above lesion level sensations, he also reported a tingling sensation in his groin/penis as his bladder reached capacity during filling while undergoing cystometry, which he reported began occurring after about 3 weeks of LT. Both subjects who completed the study with all measurements also reported ability to void during their bowel program after undergoing LT plus epidural stimulation. For example, prior to enrollment in the study, the C7 AIS B subject would leak a few drops of urine while doing his morning bowel program. Since participating in the study, he is able to void 50 to 75% of the bladder volume while doing his bowel program (estimated by amount of residual volume he collects upon catheterization afterwards). This particular finding, in addition to information regarding Viagra usage changes described above for the T4 AIS A subject, emphasizes the need for an interview component in this study, which will also provide useful data for testing new hypotheses in animal models (bedside to bench, then translation back to bedside).

3. Specific Methods.

a. <u>Interventions</u>.

Locomotor Training (LT): 80 sessions of LT will include stepping on a treadmill using body weight support (BWST) and manual facilitation in a natural position for 60 minutes each session and suspension will be provided by a harness and lift at the minimum support at which limb buckling and trunk collapse can be avoided [115]. Manual facilitation is provided to assure dynamic weight bearing equally among the legs and to enhance neuromuscular activation by providing appropriate sensory cues. Speeds will primarily be maintained within a normal speed range for walking (0.89-1.34 m/s). BWS will be continuously reduced over the course of the training sessions as the ability to bear weight on the weight bearing limbs improves and manual facilitation will be reduced as the ability to step independently improves. The treadmill speeds will be varied 25% of the time to challenge the nervous system to adapt to changes in speed (0.5 – 0.75 m/s). Subjects will be in an upright position with even weight distribution on the legs with a trainer positioned behind aiding in pelvis and

trunk stabilization, appropriate weight shifting and hip rotation during the step cycle, and ensure that the trunk and pelvis are not flexed or hyper-extended during stepping. Trainers positioned at each leg promote knee extension by applying gentle pressure at the tibial tuberosity and patellar tendon, and knee flexion and toe clearance by applying a gentle force at the medial hamstrings tendon [116].
Non-weight bearing exercise: 80 sessions of arm crank (RT300 cycle) will occur at the same intensity and duration (60 minutes) as the stepping interventions. Individuals will be sitting in their wheelchair propelling an arm crank at similar velocities to the stepping (35-55 rpm) using motor driven assistance when needed (only for those with cervical injuries whose arm function has been impaired). Resistance will be continuously increased over the course of the training sessions as the individuals are able to propel the arm crank continuously for hour.

Stand Training (weight bearing without stepping): 80 sessions of standing uses body weight support for 60 minutes each session and suspension will be provided by a harness and lift at the minimum support at which limb buckling and trunk collapse can be avoided [115].

b. <u>Procedures</u>.

A voiding diary with frequency/volume of each void and/or catheterization will be generated for two consecutive 24 hour periods once per week by using standard voiding diaries. The number of voidings, voided volume, and distribution between daytime and night-time will be obtained. In addition, urine samples will be tested once a week for the presence of blood, urobilinogen, glucose, ketones, bilirubin, protein, nitrites, leukocytes, pH, and specific gravity using DiaScreen reagent strips for urinalysis.

Urodynamics: Urodynamic study will include an initial measurement of the urinary flow (uroflow: voided volume, maximum flow rate, time to maximum flow rate and total voiding time). This will be followed by cystometry evaluation (measuring bladder pressure during filling, possible uninhibited bladder contraction and maximum cystometric capacity), with determination of the leak point pressure and postvoid residual volume when voiding is possible at the end of the study when a second uroflow can be obtained. A 12 French straight catheter will be used to empty the bladder completely and then a 7 French three way urodynamic catheter will be placed in the bladder to fill the bladder as well as to measure and record intra-vesical pressure. Another catheter with a balloon will be placed in the rectum to record the intra-abdominal pressure. Detrusor pressures will be calculated by subtracting the intra-abdominal pressure from the intra-vesical pressure. The bladder will be filled at a slow rate with body temperature water. Each subject will be asked to cough to verify intra-abdominal catheter position, and will be instructed to communicate when s/he first feels a full bladder (first sensation); when s/he first feels the desire to urinate (first urge to void); and when s/he can no longer wait to void (maximum capacity). The volume of water and bladder pressure will be recorded. Uninhibited bladder contractions will be identified. Next, the individual will be asked to empty their bladder and voiding bladder pressures recorded.

During the time when cystometry evaluation is being done, EMG needle wire electrodes will be inserted through the skin at 3 and 9 o'clock on either side of the urethra in order to record the urethral sphincter activity. Topical anesthetic cream will be used prior to insertion of the electrodes. This will evaluate coordination of the urethra and anal sphincters during the voiding phase and during possible uninhibited contraction episodes. Detrusor-sphincter dysinergia will be evaluated and classified according to the Blaivas classification into: **type 1 DSD** characterized by a crescendo increase of the sphincter activity that reaches its maximum at the peak of the detrusor contraction (as the detrusor pressure begins to decline, sudden complete external sphincter relaxation occurs); **type 2 DSD** characterized by clonic contractions of the external urethral sphincter interspersed throughout the detrusor contraction (these patients usually void with an interrupted spurting stream); **type 3 DSD**, characterized by external urethral sphincter contraction persists throughout the entire detrusor contraction (these patients void with an obstructive stream or cannot void at all) [117, 118]. EMG electrodes will also be placed during cystometry on the medial hamstrings, vastus lateralis, soleus and tibialis anterior muscles. EMG amplitudes, durations and co-activation will be calculated during filling and voiding phases of the pressure-flow study. Detailed methods have been published.

Urinary Biomarkers: Urine samples will be obtained using a sterile 12 French straight catheter that is inserted through the urethra into the bladder for cystometry. The bladder is always emptied prior to filling for cystometry recordings, so once the volume is measured, urine samples will be placed in sterile storage vials and stored at

4C for less than 3 hours. Procedures will follow similar methods established and used by multiple groups of investigators [101, 104, 119]. Briefly, samples will be centrifuged (3,000 rpm for 10 minutes) and 1 ml supernatant aliquoted into 1.5 ml tubes, with some used to determine creatine concentration. Aliquots will be frozen at -80 C for later processing. Urinary NGF and BDNF concentrations will then be determined on the thawed samples using the Emax ImmunoAssay System with specific ELISA kit per manufacturer instructions [101, 104, 119]. The concentration of NGF and BDNF in each urine sample will be extracted from a standard curve and normalized to the concentration of urinary creatine (NGF/Cr, BDNF/Cr). All samples will be run in triplicate and the values averaged.

Bladder biopsies: Dr. DaJusta will perform bladder biopsies using standard procedures at the University of Louisville Urology clinic. A random bladder biopsy will be obtained during cystoscopy, in order to capture the epithelium, basement membrane, up to the superficial layers of the detrusor muscle. The sample will be obtained in standard surgical sterile technique under local anesthesia. All layers of the bladder wall with exception of the outer serosa should be represented in the biopsy specimen (Figure 7). During this procedure, a 16 French Olympus Flexible Cystoscope (a lighted instrument used to look at the bladder) is passed through the urethra into the bladder, which may cause a feeling of slight discomfort. The bladder will be filled with normal saline through the cystoscope, producing an uncomfortable sensation, similar to a strong urge to urinate. A biopsy forceps grasper is then inserted through the cystoscope working channel into the bladder and a very small amount of bladder tissue is excised. The forceps cup has a 2 mm outer diameter but the cup itself has a 5 mm diameter. Participants may feel a pinch during the biopsy. Depending on the amount of pressure applied with this size forceps cup, a single full 1 cm biopsy sample can be obtained (i.e., plenty of tissue needed for assessments). Next, the affected blood vessels are cauterized in order to seal them and stop bleeding within the bladder wall, through a Bugbee electrode placed through the cystoscope. This may induce a burning sensation.

After the cystoscope is removed, the participant's urethra may be sore and they may report a burning sensation during urination for 1-2 days following the procedure. Viscous Lidocane will be applied through the urethra in attempt to damper this sensation. Samples will be immediately frozen in liquid nitrogen for later analyses conducted by members of the research team. To assay bladder biopsies for NGF and BDNF, frozen bladder will be homogenized and protein extracted using a lysis buffer, and subsequently run in duplicates (triplicates if tissue samples adequate) in 96-well plate antibody-sandwich ELISA with internal standard per [illegible]ay System, Promega, Madision WI, USA).

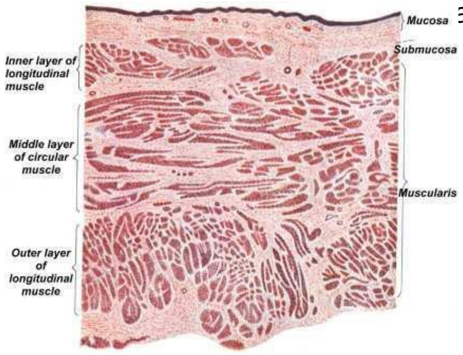

Figure 7. Layers of the bladder wall [120].

EMG activity during stepping: All methods have been published previously [47, 51]. In brief, EMG and kinematics will be acquired during stepping at four speeds (0.27, 0.45, 0.89, 1.34 m/s) using the maximum body weight load at which knee-buckling and trunk collapse can be avoided. Manual facilitation will be provided only if needed. Another stepping bout will be implemented at the load and speed where the most independence from manual facilitation occurs at the trunk, hips and legs. EMG amplitudes, durations and co-activation will be calculated. Detailed methods have been published previously.

Measures of Erectile Function: The full International Index of Erectile Function (IIEF) questionnaire is readily available in many publications [108]. It has been validated in 32 languages and can be used cross-culturally [121]. Briefly, there are 15 questions that can be divided into 5 unique domains: erectile function, intercourse satisfaction, orgasmic function, sexual desire, and overall satisfaction [108]. The Erection Hardness Grading

Scale (Grades 1-4) is defined as follows: increase in size of penis but no hardness (rigidity)as Grade 1; increase in size and slight increase in hardness (rigidity), but insufficient for sexual intercourse as Grade 2; increase in hardness (rigidity) sufficient for sexual intercourse but not fully hard (rigid) as Grade 3; fully hard (rigid) as Grade 4 [111, 112].

4. Statistical Analysis Plan. Many of the analyses involve paired comparisons of pre-training measurements (voiding frequency, bladder capacity, NGF levels, etc.) with post-training measurements (LT, stand, arm crank). We will evaluate the null hypotheses of no improvement due to training with paired t-tests. Standard F-tests will be conducted to detect variance heterogeneity, and Welch's correction subsequently applied as needed. The Wilcoxon signed rank test will be used in place of the t-test should the normality assumption be violated. Hypothesis 1C suggests an association between urodynamic parameters and EMG activity during stepping after LT. This hypothesis will be evaluated by a nonparametric correlation analysis (e.g. Spearman, Kendall correlations) comparing pre- to post-training changes in urodynamic parameters to EMG activity post-training. Power estimations using EMG co-activation data from previous human locomotor training studies indicate that a sample size of 10 per group would achieve a significant difference at the 0.05 level between the groups with a power of 80%.

Hypotheses 3A-3D are specific to Group 2 (LT + epidural stimulation) and each involves comparing the change in outcome variables over 80 LT sessions to changes over 80 stand + epidural stimulation sessions then 80 LT + epidural stimulation sessions. We will use paired t-tests to compare the improvement over 80 sessions of LT to the improvement over 80 sessions of stand + epidural stimulation, and like-wise for 80 sessions of stand + epidural stimulation to the improvement over 80 sessions of LT + epidural stimulation. Welch's correction and the Wilcoxon signed rank test will be applied as described above.

Descriptive statistics for each outcome will be calculated for each study group at each assessment. In particular, we will generate mean and standard error estimates for each outcome variable pre-training, post-training, and for the pre-post training difference, so that these quantities can be used in planning future research. All hypothesis tests will be conducted at the .05 level. It is possible that results derived from Specific Aim 3 (study of LT + epidural stimulation) may understate the effect of LT + epidural stimulation, as the effect of epidural stimulation added to LT may be partially masked by the gains experienced over the initial 80 sessions of LT received.

5. Limitations and potential pitfalls. Time since injury will vary by years. It is difficult to confine time since injury to within a few months and also recruit a sufficient number of SCI research participants. It is possible that the time since injury would influence the outcomes. However, we have shown neural changes in individuals' even decades after clinically complete SCI [113] with the most significant change in bladder function reported from an individual 8 years post injury. A key element of risk is that we have relied on patient reports of bladder improvements coincident with the intense LT. Improved bladder function, even incremental, would have a dramatic impact on health and quality of life for those suffering the lifelong consequences of neurologic injury. Also, individuals will vary in terms of their continence and voiding as well as sexual function, including opportunities for sexual activity, types of stimulation that work, and positioning problems with paralysis. For these reasons, every participant has pre- and post- measurements for all aspects of the study, so that each subject can also serve as their own control (in addition to quantification of group trends). There may exist a high variability in the magnitude of outcomes. However, if a significant number of individuals recover even some bladder function it would have a tremendous impact on their quality of life and potentially reduce infections and longer term bladder complications.
