# Supplementary material for: Improvements in bladder, bowel and sexual outcomes following task-specific locomotor training in human spinal cord injury
Source: PLoS One. 2018 Jan 31;13(1):e0190998. doi: 10.1371/journal.pone.0190998 (PMC5791974; doi:10.1371/journal.pone.0190998)
Supplement: S2 File — University of Louisville IRB approval document for the study “Effects of activity dependent plasticity on recovery of bladder and sexual function after human spinal cord injury”. (PDF) [file pone.0190998.s002.pdf]

UNIVERSITY OF LOUISVILLE

Human Subjects Protection Program Office
MedCenter One – Suite 200
501 E. Broadway
Louisville, KY 40202-1798
Office: 502.852.5188 Fax: 502.852.2164

DATE: September 03, 2014

TO: Susan J Harkema

FROM: The University of Louisville Institutional Review Board

IRB NUMBER: 14.0062

STUDY TITLE: **Effects of activity dependent plasticity on recovery of bladder and sexual function after human spinal cord injury**

REFERENCE #: 336882

DATE OF REVIEW: 09/03/2014

IRB STAFF CONTACT: Name: Tammy Peek
Phone: 852-4535
Email: tammy.peek@louisville.edu

This study was reviewed by the Full Board on 08/21/2014 and approved contingent upon changes to the study documents. The changes were received and reviewed by expedited review on 09/03/2014. **This study now has final IRB approval from 08/21/2014 through 08/20/2015.**

Documents/Attachments reviewed and approved:

Title	Version Number	Version Date	Outcome
Appendix A-Epidural Stimulation Protocol dated 6.21.14	Version 1.0	08/26/2014	Approved
14.0062 AUAF_SUI_Bladder_Diary	Version 1.0	07/30/2014	Approved
ERECTILE QUESTIONNAIRE	Version 1.0	05/14/2014	Approved
FSFI QUESTIONNAIRE	Version 1.0	05/14/2014	Approved
14.0062 RO1_ResearchStrategy Protocol dated 8.20.14	Version 1.1	08/20/2014	Approved
LT ICF & RA Bladder-Sex Function dated 8.22.14	Version 1.1	08/22/2014	Approved
Consent & RA Bladder-Sex Function dated 8.22.14	Version 1.1	08/22/2014	Approved
Partial Waiver Bladder-Sex Function dated 8.22.14	Version 1.1	08/22/2014	Approved
Epi ICF & RA Bladder-Sex Function dated 8.22.14	Version 1.1	08/22/2014	Approved

Site Approval

If this study will take place at an affiliated research institution, such as KentuckyOne Health, Norton Healthcare or University of Louisville Hospital, permission to use the site of the affiliated institution may be necessary before the research may begin. If this study will take place outside of the University of Louisville Campuses, permission from the organization should be obtained before the research may begin. Failure to obtain this permission may result in a delay in the start of your research.

Privacy & Encryption Statement

The University of Louisville's Privacy and Encryption Policy requires such information as identifiable medical and health records: credit card, bank account and other personal financial information; social security numbers; proprietary

research data; dates of birth (when combined with name, address and/or phone numbers) to be encrypted. For additional information: http://security.louisville.edu/PolStds/ISO/PS018.htm.

Implementation of Changes to Previously Approved Research

Prior to the implementation of any changes in the approved research, the investigator will submit any modifications to the IRB and await approval before implementing the changes, unless the change is being made to ensure the safety and welfare of the subjects enrolled in the research. If such occurs, a Protocol Deviation/Violation should be submitted within five days of the occurrence indicating what safety measures were taken, along with an amendment to revise the protocol.

Unanticipated Problems Involving Risks to Subjects or Others (UPIRTSOs)

In general, these may include any incident, experience, or outcome, which has been associated with an unexpected event(s), related or possibly related to participation in the research, and suggests that the research places subjects or others at a greater risk of harm than was previously known or suspected. UPIRTSOs may or may not require suspension of the research. Each incident is evaluated on a case by case basis to make this determination. The IRB may require remedial action or education as deemed necessary for the investigator or any other key personnel. The investigator is responsible for reporting UPIRTSOs to the IRB within 5 working days. Use the UPIRTSO form located within the iRIS system to report any UPIRTSOs.

Continuation Review Requirements

You are responsible for submitting a continuation review 30 days prior to the expiration date of your research study. Investigators who allow their study approval to expire have committed significant non-compliance with federal regulations. Such lapses may require reporting to federal agencies, a program audit by compliance auditors to ensure that subjects were not enrolled during the expired period, and may lead to findings of serious and continuing non-compliance if expiration were to occur a second time.

1099 Information (If Applicable)

As a reminder, in compliance with University policies and Internal Revenue Service code, all payments (including checks, gift cards, and gift certificates) to research subjects must be reported to the University Controller's Office. Petty Cash payments must also be monitored by the issuing department and reported to the Controller's Office. Before issuing compensation, each research subject must complete a W-9 form. For additional information, please contact the Controller's Office at 852-8237 or controll@louisville.edu

If you have any questions, please contact the HSPPO at (502) 852-5188 or hsppofc@louisville.edu

Thank you for your submission.

Sincerely,

Julie L. Goldman, MD
Vice Chair, Biomedical Institutional Review Board
